# Pit lakes from Southern Sweden: natural radioactivity and elementary characterization

**DOI:** 10.1038/s41598-020-70521-0

**Published:** 2020-08-13

**Authors:** J. Mantero, R. Thomas, E. Holm, C. Rääf, I. Vioque, C. Ruiz-Canovas, R. García-Tenorio, E. Forssell-Aronsson, M. Isaksson

**Affiliations:** 1 Department of Radiation Physics, Institute of Clinical Sciences, Sahlgrenska Academy at University of Gothenburg, 413 45 Gothenburg, Sweden; 2 Department of Applied Physics II, ETSA, University of Seville, 41012 Seville, Spain; 3 Medical Radiation Physics, Department of Translational Medicine ITM, Lund University, Malmö, Sweden; 4 Department of Earth Sciences & Research Center on Natural Resources, Health and the Environment, University of Huelva, 21071 Huelva, Spain; 5 Spanish National Accelerator Centre (CNA), University of Seville, 41092 Seville, Spain

**Keywords:** Freshwater ecology, Environmental monitoring

## Abstract

Natural radioactivity in the environment is a field gaining more attention in last decades. This work is focused on the study of natural radioactivity complemented with elementary characterization at former non-uraniferous mining areas in Sweden. This aim is addressed through the study of mining lakes, called pit lakes, which are water bodies generated after opencast mining. Environmental matrices (water, sediments and rocks) from 32 Swedish pit lakes, commonly used for recreational purposes were radiometrically characterized via alpha (^238^U, ^234^U, ^232^Th, ^230^Th, ^210^Po isotopes) and gamma spectrometry (^238^U and ^232^Th series radionuclides). Additionally, ambient dose rate equivalent in the immediate surrounding of each pit lake was quantified. Physico-chemical parameters (pH, specific conductivity, dissolved oxygen, oxidation–reduction potential) and elemental composition (major and trace elements by ICP-MS) were analysed in water samples and elementary composition of sediments/rocks was measured by XRF and SEM–EDX in some specific cases. A non-negligible number of pit lakes (26%) with enhanced U levels in water was found. At some sites, rocks contained up to 4% of U in areas with high degree of interaction with local population. Concerning the elementary perspective, another popular site (due to its turquoise water) was found to have elevated dissolved heavy metal levels. Results obtained in this work prove that measurement of natural radioactivity is another component that should be included in routine analysis of characterization in mining areas, especially if restauration of post-mining sites is intended for human recreational.

## Introduction

Mining activities in Sweden, the major metal mining country in the European Union, involving 63% of iron ore production, Zn (22%), Pb (20%) and Ag (17%) in 2014^1^, imply the generation of enormous quantities of mining wastes. Historically, more than 2,700 mines gather around 30,000 sites that have been minor mines and quarries according to the Geological Survey of Sweden (only 15 active mines in 2015)^2^. Many of these sites were opencast mines to exploit sulfide, limestone, clay, etc. It is noteworthy that open-pit mining has increased substantially over the past two decades due to improvement of metallurgical techniques that enable metal extraction from low-grade ores^3^. During exploitation by open-pit mining, the water table is suppressed to avoid the flooding of active mines. However, when mining activity ceases, the water table recovers its original position, flooding the open pits, and giving rise to mine pit lakes.

The geochemistry of pit lake waters can vary enormously, depending on several factors such as local geology, hydrology or climate^4–7^. A significant example is the case of sulfide mine pit lakes, where high/very high concentration of heavy metals in waters can be found due to the generation of acid mine drainage (AMD) processes^7^. An important feature of AMD is that the sources of pollution can be active for years or even centuries after mine closure^8^. However, other anthropogenic factors may have a significant impact on lake waters. For instance, the atmospheric deposition of acidifying compounds released mainly by industry led to severe acidification of lakes and streams^9^. Liming has been extensively used in Sweden since the 1970s to offset the negative consequences of acidification^10^. In total, around 8,000 lakes have been limed at least once and about 20 M€/year has been devoted to liming of surface waters in Sweden^11^. The major goals for the Swedish liming program were to keep alkalinity above 0.05 meq/l and pH above 6.0 in order to protect existing flora and fauna and to let species recolonize. Liming causes the transference of metals (i.e. Al, Cd, Co, Ni and Zn) from the water column to the lake sediments due to increase of pH values. However, the fluxes of metals can change if reacidification takes place, leading to increasing metal levels in the water column. Despite the decrease of acidifying compound emissions and the signs of surface water recovery primarily observed in Scandinavia, critical loads of acidifying compounds are still being exceeded in southern Sweden by a factor of between two and five^12^. Apart from the potential impact on the ecosystem, many of these lakes are nearby surrounded by populated areas and most of them are used for recreational purposes (swimming, fishing, diving, etc.), and hence the potential risk from a human perspective becomes a relevant issue to address. Therefore, many of the selected sites in this work come from a diver’s forum in Sweden^13^. For these reasons, the quality of these waters should be studied in order to assess the environmental and human health risks associated with these activities.

Another perspective of the physico-chemical characteristics of pit-lake systems tackled in this paper is their radiological environment, which is determined by means of radiometric assays. These techniques/assays are mainly reserved to U mining sites, where we can find pit lakes with ^238^U activity concentrations in the range of 15 to 40 Bq/L in Kazakhstan^14^, Tajikistan^15^ or Brazil^16^. However, in a former copper mine pit lake 10.5 Bq/L of ^238^U was measured, which is close to the typical ranges of U mining pit lakes^17^. In Europe, the mean geochemical background U concentration in continental surface water is in the order of 0.889 µg/L (11.1 mBq/L of ^238^U)^18^.

Mining activities commonly increase the mineral surface area to air and water and potentially expose more minerals to weathering. Thus, mining operation can lead to the release of natural radionuclides originally contained in the host rock to the environment. Previous studies on Spanish pit lakes show that, apart from high levels of heavy metals, enhanced levels of natural radionuclides can occur at these sites^19^. Activity concentrations reported in Spanish pit lakes ranged from 14 to 1,110 mBq/kg of ^238^U in sulfide mine pit lakes (the higher value exceeding 100 times the European mean U surface water activity concentration). This enhancement was directly related to the AMD process, and water samples had pH values from 2.2 to 2.7. However, enhanced levels on natural radionuclides were also found in phosphate/carbonate mining pit lakes in the Moulouya district mining in Northern Morocco^20^. In this recent work, surficial water with ^238^U activity concentration ranging from 235 to 1,027 mBq/kg were found in pit lakes with pH values of 9.2–9.6. Alkaline pH values and elevated bicarbonate concentrations in oxidized surface waters favour the stabilization and mobilization of uranium as uranyl-carbonate complex^21,22^. In contrast, the predominant species in acid, oxygenated waters are the uranyl ion and the uranyl-sulphate complex^21^. In this sense, liming of acidified lakes causes a depletion of metals from the water column to the sediment, but may increase the mobility of natural radionuclides. However, to our knowledge, this issue has not been properly addressed until now.

Therefore, the main goal of this work is to assess the distribution of radionuclides and elementary characterization of non-uraniferous pit lakes from southern Sweden, producing a database for further and deeper studies on specific sites.

## Material and methods

Several mining resources and databases were checked to select the sampling sites among hundreds of possibilities^2^. The survey cohort consisted of a subgroup of 23 sites containing 32 pit lakes that were covered within three sampling campaigns, performed during April, July and October 2015. In the supplementary file, more detailed information is provided about the sampling site locations including pictures of every sampled pit lake (Supplementary material: Table S1 and Pictures 1–34).

### Sampling sites

**Figure 1:**
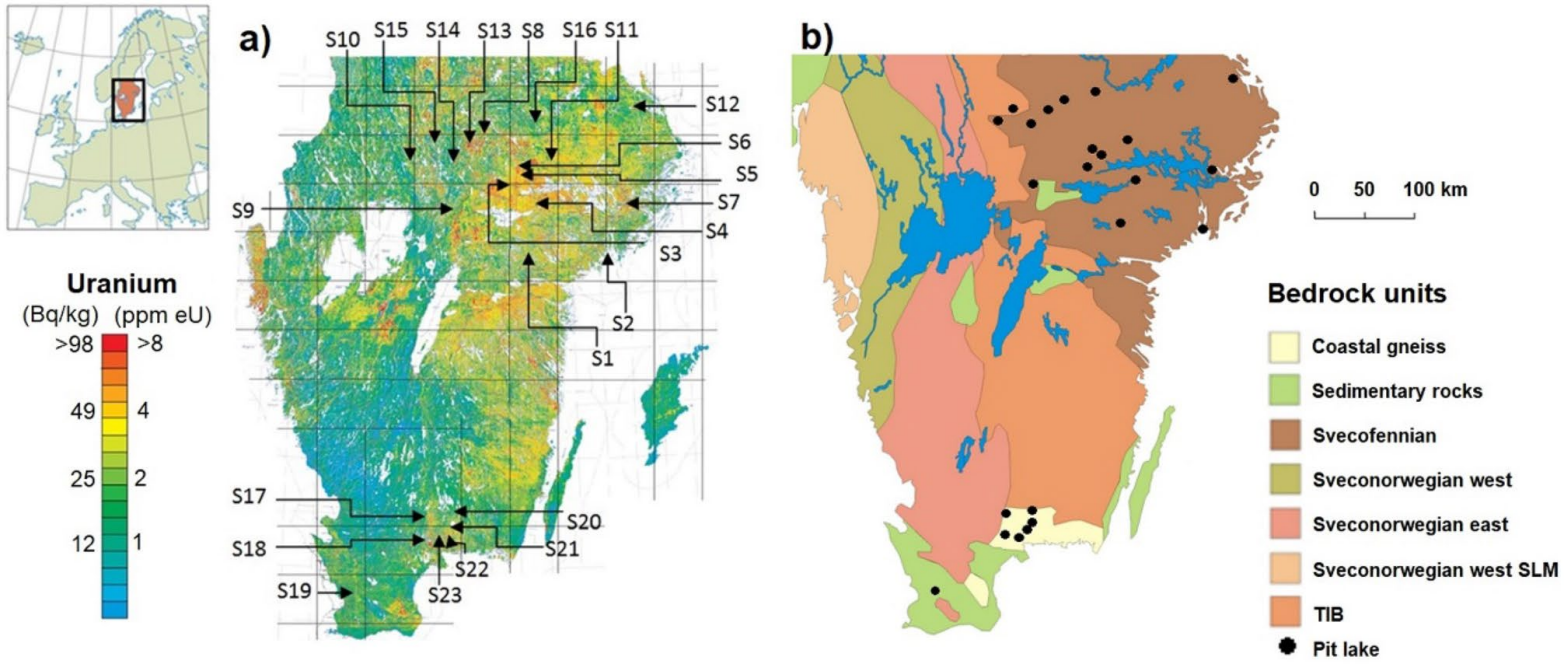
(**a**) Location of sampling sites shown at a map of U-238 activity concentration from airborne gamma measurements originally supplied by SGU^30^ and modified using the graphic editor Paint included in Microsoft Windows 10. (**b**) Bedrock map, originally from^50^ and modified by using Corel Draw 10 software.

The map with the location of the 23 examined mining sites (with totally 34 pit lakes) in southern Sweden is shown in Fig. 1. A preliminary screening was performed after overlapping the sampling location map provided by a website of pit lakes used for recreational purposes^13^ with radiometric U airborne maps provided by SGU (Fig. 1a). Most of the sites were randomly distributed in areas where ^238^U in rocks had a concentration of ca 4 ppm or higher.

Concerning the geological perspective, the bedrock materials of southern Sweden are mainly composed of Precambrian crystalline rocks, belonging to the Baltic shield, which is usually divided into five geological provinces^23^. Of them, only the Svecofennian province and the more recent Sveconorwegian province are found in the west and east, respectively, of southern Sweden. Between both provinces, the Transscandinavian Igneous Belt (TIB) is also found. Bedrocks of Svecofennian province are mainly composed of metasedimentary and metavolcanic rocks and several generations of granitoids, and may host important ore deposits (i.e. iron and sulfide ores). The TIB consists of largely undeformed granitoids and associated porphyries. It stretches from Småland in southern Sweden through Värmland and western Dalarna (where it is partly covered by Jotnian sandstone) and then continues under much of the Caledonian mountain chain up to northern Scandinavia. The Sveconorwegian province may be further subdivided into an eastern and a western segment, which represent different episodes of formation and have been subject to extensive metamorphisation^24^. Among these materials, younger rocks with completely different formation histories can be found such as dykes of diabase or similar minerals, but more importantly some sedimentary rocks, mainly formed by limestone, shale and slate, with flat-topped mountains covered by diabase (Fig. 1b).

### Matrices

Surface water was sampled at each pit lake (n = 34 water samples) in 5 L polyethylene jerry cans (FISHER, USA). A first cleaning with distilled water was applied to each bottle in the field, followed by a rinsing with water from the pit lake before sampling. Samples were manually collected from the shoreline. A second 45 mL aliquot was also sampled for chemical composition determination and it was immediately acidified with concentrated HNO_3_ (65%) to pH < 2 to prevent precipitation and adsorption reactions within the container walls. A blank sample was also prepared by adding 65% HNO_3_ to 45 ml of distilled water. These samples were filtrated in the lab with a qualitative filter paper (35–40 µm pore size) for particle retention (WHATMAN, England). The main aim at this stage of the project was to measure the total elementary content in water (not only the dissolved fraction where a 0.45 µm pore size is used). The 5 L aliquot, intended for radiometrical analysis, was also acidulated to pH < 2 and was not filtrated in order to enable an assessment of the total concentration of radionuclides.

Sediments were collected from the shoreline (20–30 cm depth), taking care to sample only the top-1-cm layer. Only 10 sites allowed sampling of sediments with a total of 14 samples. When possible, different spot samples (~ 0.5 kg wet weight in each spot) along the shoreline were collected to make a composite sample from the entire lake. Most of the pit lakes are small in size with water surfaces below 1 hectare, and for that reason the sampled sediments could be considered as representative of the pit lake. Sediments were packaged in zip plastic bags. Once in the laboratory samples were homogenized, dried at 80 ºC, milled and finally sieved to 1 mm size for chemical composition (X-ray fluorescence) and radiometric determinations (alpha and gamma spectrometry).

In addition, some rocks were also sampled on sites where elevated ambient dose rate equivalent was recorded. The rock samples were milled and sieved to the same size as for the sediments (1 mm).

### Techniques and measuring systems

Methodology and systems used during this work is described with more detail^25^ together with the quality assurance program. As a consequence, only a brief description will be provided about methodology in this section.

#### Physico-chemical parameters in water

Physico-chemical parameters as temperature, pH, specific conductivity (SC), oxidation–reduction potential (ORP), total dissolved solids (TDS) and dissolved oxygen (DO) were measured immediately in the field with a multiparametric probe. During the first sampling a Professional Plus multiparametric meter (YSI, USA) was used, while for consecutive samplings, a MS5 multiparametric meter (HYDROLAB, USA) was used. These instruments were calibrated with certified standards solutions from the probe suppliers (pH, SC, ORP and TDS) before each sampling campaign.

#### ICP-MS

Elementary composition of samples with major (Na, Mg, P, S, K, Ca) and trace (Fe, Mn, Cr, Cu, Zn, As, Sr, Ba, Pb, U and Th) elements were determined by Inductively Coupled Plasma-Mass Spectroscopy, model Agilent 7500c. Two aliquots per sample of the 45 ml water sample vial were diluted a factor 50 before being measured by ICP/MS. Sample introduction was performed with a PFA (perfluoroalkoxy) microflow auto-aspirating nebulizer combined with a double-pass spray chamber (AGILENT TECHNOLOGIES, Japan). A Multi-element Calibration Standard solution in 5% nitric acid media (provided by Agilent Technologies) containing: Al, Sb, As, Be, Cd, Cr, Co, Cu, Pb, Mn, Mo, Ni, Se, Tl, Th, U, V and Zn was properly diluted and used in every analysis sequence which also included blank samples to control the performance throughout the measurement sequence. The relative uncertainty (95% confidence level) for these elements ranges between 15 and 20%.

#### Alpha spectrometry (U, Th and Po isotopes)

Environmental samples (water/sediment/rock) underwent three main processes: pre-concentration, separation and finally the alpha source preparation. Depending on the matrix, the pre-concentration stage differs, while separation and alpha source preparation procedures were the same for water and sediment/rocks.

During pre-concentration stage, and after the spiking of the selected aliquots with a known amount of tracers (^232^U, ^229^Th and ^209^Po) in the case of waters, 0.5 L were submitted to iron hydroxide precipitation while in the case of sediments/rocks, 1 g of sample were microwave digested with 40% HF plus aqua regia. Once the samples were digested, the solutions underwent also an iron hydroxide precipitation process. From the water and sediment/rocks iron precipitates the Th, U and Po fractions were isolated by combining liquid–liquid separation and extraction chromatography techniques. The isolated fractions of U and Th were then electroplated onto steel discs while instant deposition onto Cu discs was applied for Po isotopes. Complete description about radiochemistry used in these work can be found in^25^.

Alpha sources were measured with Passivated Implanted Planar Silicon detectors from Canberra in an Alpha-Analyst system and ULTRA Ion-Implanted-Silicon Charged-Particle detectors from Ortec in an Alpha Ensamble system. Due to the low activity concentration levels in environmental samples, the acquisition time was chosen to 200,000 s to obtain relative stochastic uncertainties in activity concentration around 5% and , in general, a minimum detectable activity (MDA) below 0.5 mBq for the different U, Th and Po isotopes.

#### Gamma spectrometry

Sediments/rocks, were dried, grounded and, after 1 mm sieving, packed in a plastic cylindrical 35 mL geometry with only 10 mm height to minimize self-absorption effects. As reference material for photopeak efficiencies, two matrices were used: IAEA-RGU-1 for ^238^U- and ^235^U-series radionuclides and IAEA-RGTh for the ^232^Th-series radionuclides. As routine methodology, self-absorption correction was applied according to^26^.

NORM radionuclides from ^238^U series studied were ^210^Pb, ^234^Th, ^226^Ra (determined by secular equilibrium using ^214^Pb and ^214^Bi) and also ^234^Pa^m^ (measured when possible due to its very low gamma yield). From ^232^Th series: ^228^Ra was obtained via ^228^Ac and ^228^Th via ^212^Pb, ^212^Bi and ^208^Tl. Additionally, ^40^K and the anthropogenic ^137^Cs were measured. Gamma measurements were performed in an extended range germanium coaxial detector (XtRa) of 37.1% relative efficiency. For a 200,000 s acquisition time, this system provides MDA values ranging from 1.5 to 5 Bq/kg for radionuclides from the ^238^U and ^232^Th series, ~ 15 Bq/kg for radionuclides in the ^235^U-series, ~ 10 Bq/kg for ^40^K and around 1 Bq/kg for ^137^Cs.

#### X-ray fluorescence (XRF)

The elementary composition (mainly trace elements as Fe, Mn, S, Ba, Pb, Zn, Sr, Cr, Cu, As, Th, U) in sediments and rocks were performed at the X-Ray Laboratory of the University of Seville by wavelength Dispersive X-Ray Fluorescence (WDXRF). Around 0.1 g of sediment/rock is mixed with 0.01 g agglomerant (LICOWAX) and is pressed for 1 min to 200 kN on top of a boric acid mould. Finally a 40 mm diameter cylinder of boric acid with a 10 mm inner centered cylinder containing the sample is produced and measured with an AXIOS system (MALVERN PANALYTICAL, United Kingdom /Netherlands). Reference materials were used: MBL-1 (basalt), GYP-B (ore SO_4_), JCRM R041 (Mullite) and NCS DC71305 (rock) to validate this methodology. Relative uncertainty (with 95% confidence level) ranges from 1.7% for S until 30% for P, averaging 11.5% for all elements. Detection limits range from 1 ppm for Th until 100 ppm for Mn averaging 22 ppm.

#### Ambient dose rate equivalent

Two independent external gamma dose meters (Rados SRV 2000 Compensated GM-tube with an energy range: 50 keV–3 meV and a dose rate range: 0.05 µSv/h–10 Sv/h), calibrated for ambient dose rate, H*(10), were placed 1 m above the ground at different places around each site during the sampling time. Each system provided an average value over 15 to 20 min measurement. The result used to represent the final estimate of the ambient dose rate at the site was the average of several measurements at different spots. The two dose rate meters were quality checked before use.

#### Scanning electron microscopy-energy dispersive X-ray spectroscopy (SEM–EDX)

In some particular cases where rocks were found with enhanced levels of natural radionuclides, SEM–EDX was used for a morphological characterization and for local elementary composition. For that purpose, a JEOL 6460LV scanning electron microscope was used, equipped with acquisition of digital images in both secondary (SEI) and backscattered (BEI) electron imaging modes (maximum resolution 3.5 nm). This device was coupled to an EDX microprobe and fitted with an ATW2 beryllium window (resolution 137 eV at 5.9 keV). The semi-quantitative analysis was performed using the Oxford INCA software.

#### Data uncertainties

All reported uncertainties/error bars are shown with k = 1 criteria. Regarding ICP-MS measurements, 20–25% uncertainty is reported (semi-quantitative analysis), while XRF provides 15–20% uncertainty. Gamma spectrometry precision is in the range of 10–15%, while it is 7–10% for alpha spectrometry. These uncertainties depend on how close the measured values are to the MDA of the technique. Software used for statistical treatment of data was OriginPro 8.0 (ORIGINLAB, USA).

#### Sediments as markers of pollution

In order to use the elementary composition measured in sediments to identify any potential polluted site, diverse methods were found in the literature. There are several approaches to study the interaction between the water column and sediments by parameters such as enrichment factor (EF) or index of geoaccumulation (Igeo)^27,28^. However, we decided to use the method proposed by Håkanson^29^, developed for lakes in Central Sweden, the same area considered in this survey. In accordance with Håkanson’s model, sediment composition can be used as a diagnostic tool for water pollution control. A parameter defined as *degree of contamination* (C_d_) is assessed based on relative elementary composition in lake sediments to reference level based on 8 elements (Hg, Cd, As, Cu, Pb, Cr, Zn and PCB, polychlorinated biphenyl), and following the equation:

1 $${C}_{d}=\sum_{i=1}^{8}{C}_{f}^{i}=\sum_{i=1}^{8}\frac{{C}_{0-1}^{i}}{{C}_{ref}^{i}}$$

where ${C}_{0-1}^{i}$ is the concentration of the i-th element in the sediment (sampled from 0 to 1 cm layer) and ${C}_{ref}^{i}$ is the *standard preindustrial reference level* determined from various European and American lakes. ${C}_{f}^{i}$ is defined as *contamination factor* (C_f_) of the i-th element.

Elementary composition of 14 composite sediment samples will be included in Eq. (1) to assess both degree of contamination and contamination factors on each site.

## Results and discussion

### Elemental and radiometrical characterization of surface water

#### Physico-chemical parameters of surface water

**Figure 2:**
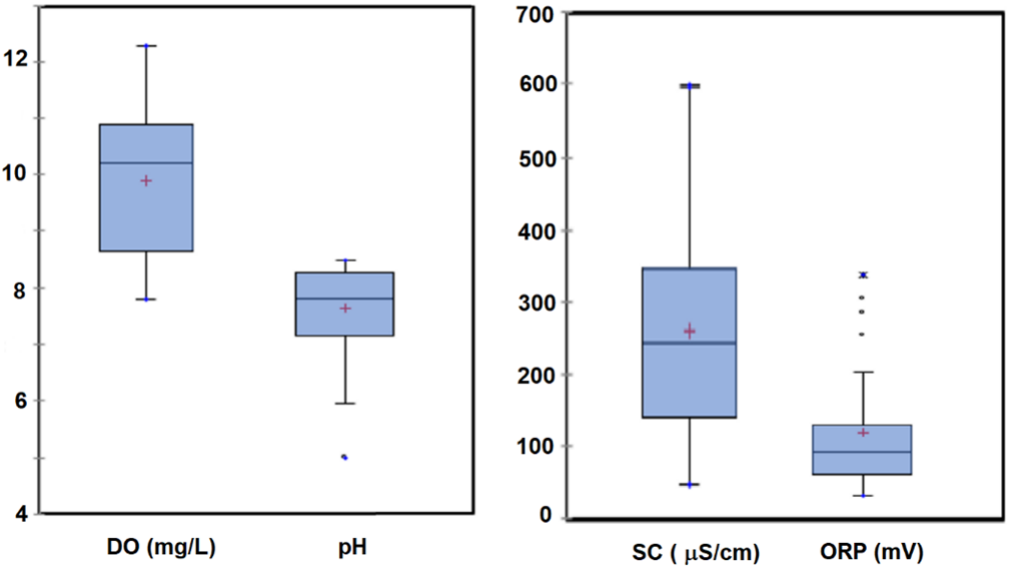
Box-and-whisker plots of physico-chemical parameters (i.e. pH, specific conductivity (SC), oxidation–reduction potential (ORP) and dissolved oxygen (DO)) in pit lake surface water samples. The height of the box shows the interquartile range, which contains 50% of the values, while the horizontal line inside the box shows the median value and the red cross denotes the mean value. The whiskers are lines that extend from the box to the highest and lowest values excluding outliers (o) and extremes (*). In this sense, outliers represent those values being between 1.5 and 3 times larger than the length of the box from its upper or lower border while extreme are those greater than 3 times such value.

**Figure 3:**
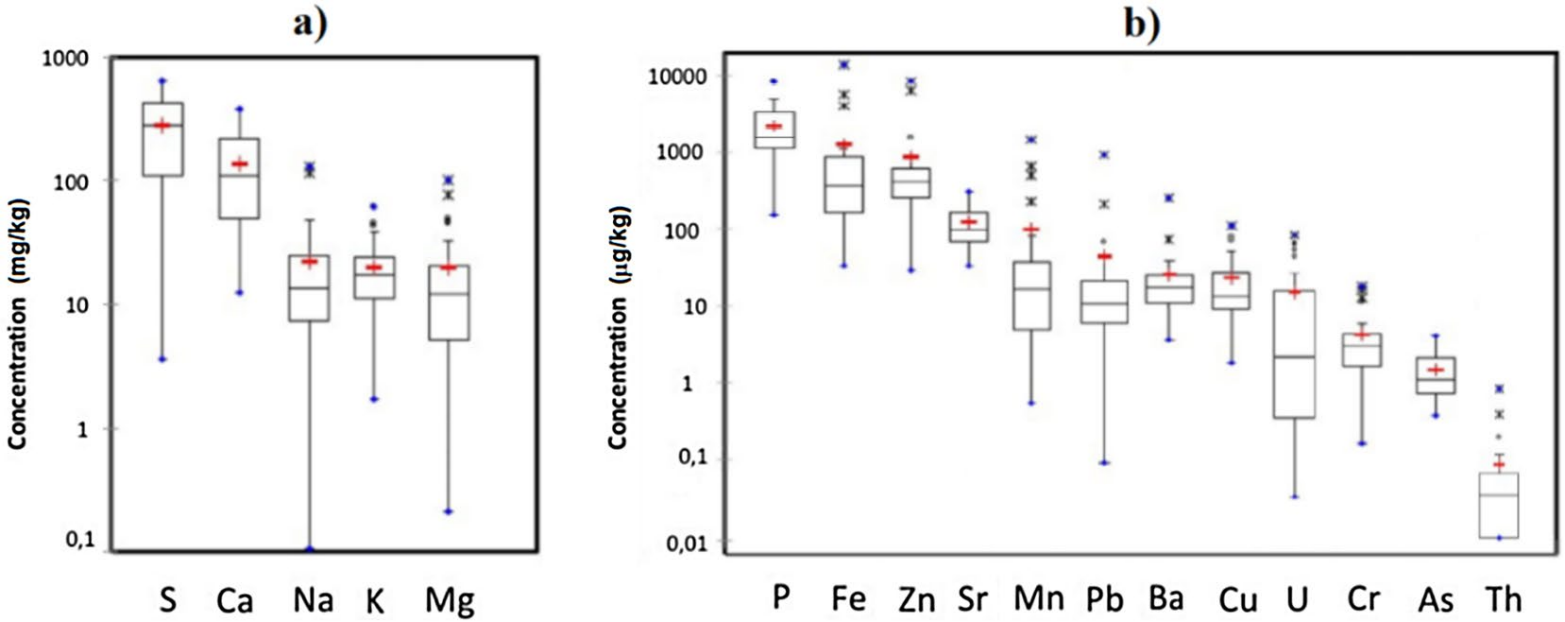
(**a**) Concentration of elements in mg/kg and (**b**) in µg/kg of surficial water samples from the studied pit lakes, shown as box-and-whisker plots (refer to Fig. 2 for explanation) and sorted out by mean values.

The physico-chemical parameters (i.e. pH, SC, ORP and DO) in surficial water samples from the 23 sampling sites are shown in Fig. 2 (Raw data in Table S2 from supplementary material). Results show low SC values of 47–597 µS/cm (average value of 260 µS/cm). The maximum SC values stemmed from Site 4 (due probably to the presence of sulfide-bearing schists in bedrocks outcropping in the drainage area according to the SGU local bedrock map^30^. Average pH values of 7.6 were observed in the studied lakes, with an interquartile between 7.1 and 8.2. Such high values could be due to the Swedish liming program initiated in 1977 to counteract the anthropogenic acidification observed in Swedish surface waters^31^. However, the minimum value of 4.9 was observed at Site 14, due to sulfide mining activities in this site, a derelict silver mine operated discontinuously from 1,483 to 1,900, leading the formation of a pit lake of around 240 m depth. The oxidation of galena and other sulfides found originally in this site may have caused such low pH values. The average ORP value in lake waters was 95 mV (interquartile range of 41 to 100 mV; Fig. 2), although a maximum value of 300 mV was observed at Site 4, where sulfide-bearing schists appear to outcrop in the drainage basin. Water samples were well oxygenated with DO values from 7.8 to 12 mg/L (65–99% of saturation) and average 9.9 mg/L. Such high concentrations of dissolved oxygen together with the low values of total phosphorous (average values of 2.5 mg/L, interquartile range of 1.3 to 3.8; Fig. 3), seem to indicate an oligotrophic nature of lakes studied.

#### Major elements and trace elements in surface water

The content of dissolved elements in lakes is primarily controlled by rock weathering, atmospheric precipitation and evaporation-precipitation processes^32^. Ca and S are the main elements in disolution; in studied pit lakes average values of 140 and 280 mg/L of Ca and S (interquartile range of 50–220 mg/L and 110–420 mg/L), respectively, were recorded (Fig. 3a). Raw data to produce Fig. 3a,b) can be found as Table S3 in supplementary material. The main sources of S may be dry deposition and the acid rain in industrialized and urban areas; i.e. most lakes studies are found close to large cities (Stockholm, Örebro, Norköpping, etc.) and to a lesser extent the oxidation of minor amounts of sulfides present in the drainage catchment, while the high concentrations of Ca found in these lakes should be related to liming and to a lesser extent, to the dissolution of carbonates in old marble quarries. Compared to these elements, the concentration of others such as Na, Mg or K is noticeably lower; around 20 mg/L were observed (Fig. 3a). However, maximum values exceeding 100 mg/L of Na and Mg were found in Site 4 and Site 11, respectively. The latter site was a former quarry where marble (calcium-magnesium carbonate) was exploited. The intense water–rock interaction after flooding may have released significant concentrations of Mg to the water column.

Concerning trace elements, the most abundant is Fe with average values of 1,200 µg/L, followed by Zn (860 µg/L), Sr (120 µg/L), Mn (100 µg/L), Pb (38 µg/L), Cu (23 µg/L), Ba (22 µg/L), Cr (4.3 µg/L) and As (1.5 µg/L). The maximum values of Fe and Mn were observed at Site 13 (13,700 and 1,460 mg/L, respectively; Fig. 3b), probably due to the presence of colloidal material rich in Fe and Mn passing through the pore filter. This fact would also explain the high concentration of other trace metals such as Zn (8,400 mg/L). On the other hand, the average concentration of Th and U are 85 ng/L and 14 µg/L, respectively, although maximum values of 750 ng/L and 68 µg/L were observed. Such different values between U and Th may be related to differences in mobility of U and Th species despite that granites, the most abundant rock in the studied area, are mostly enriched in Th over U^33^. This enrichment of Th over U also applies to alkaline rocks (K or Na >  > Ca) according to Dill^34^.

#### Alpha spectrometry of surface water

##### Uranium radioisotopes in surface water

**Figure 4:**
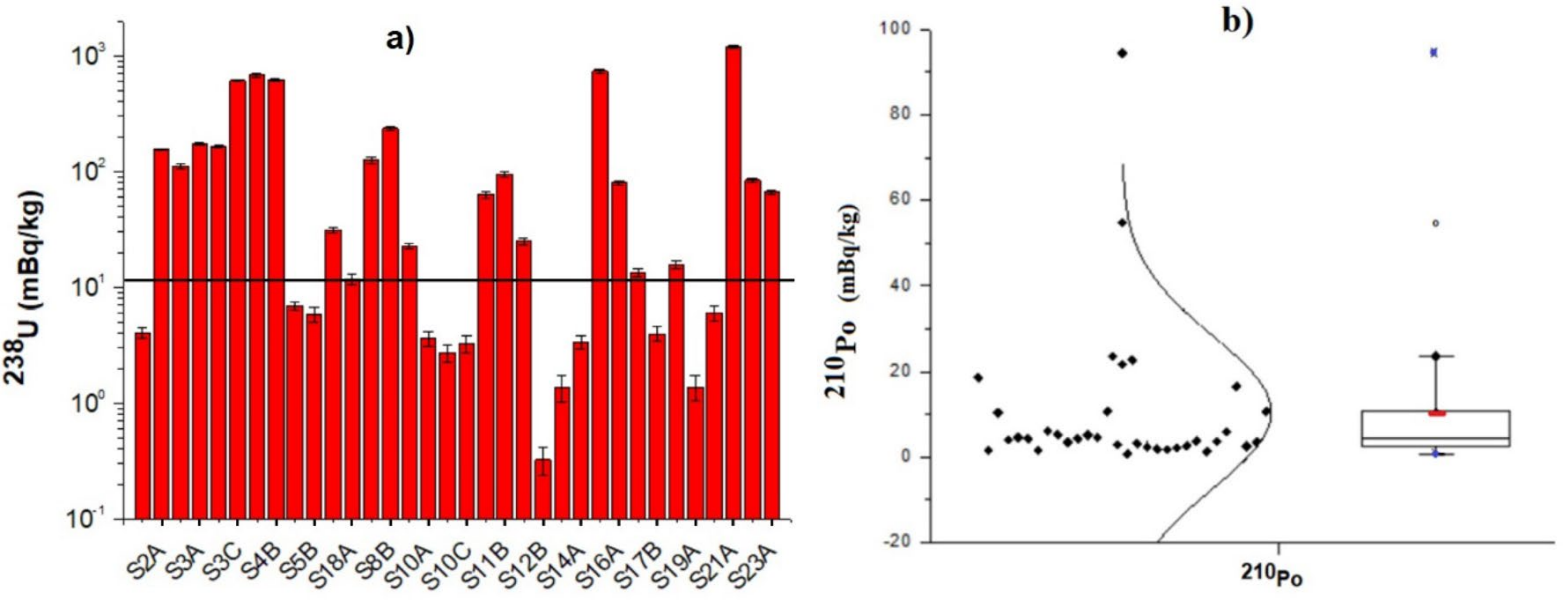
(**a**) ^238^U activity concentration levels in surface water samples from the studied pit lakes in southern Sweden. The horizontal line shows the mean geochemical background^18^. (**b**) ^210^Po activity concentration in surface water samples from pit lakes, presented together with its normal distribution and a box-and-whisker plot. Raw data can be found in Table S4.

A range from 0.3 to 1,183 mBq/kg with a mean value of 156 ± 272 mBq/kg (mean ± standard deviation) was found for ^238^U isotopes (Fig. 4a). Raw data to produce Fig. 4a,b and Table 1 can be found as Table S4 in supplementary material. These values could be compared with the geochemical background values of ^238^U for continental surface waters ranging from 0.02 to 266 mBq/kg and a mean value of 11 ± 21 mBq/kg^18^. The 72% of the sites had levels above the mean background value, so there is in general an enhancement of ^238^U level in pit lakes in southern Sweden. Furthermore, one natural lake was sampled at the beginning of each sampling campaign to be used as “reference value” in comparison with pit lakes. The ^238^U in a subset of 3 natural lakes ranged from 0.34 to 11 mBq/kg with mean 5.0 ± 5.2 mBq/kg), which is in the order of the environmental background value.

**Table 1:** Summary of activity concentration of U, Th and Po isotopes from ^238^U and ^232^Th series in pit lake surface water samples (n = 34).

	Activity concentration (mBq/kg)				Concentration ratios		(mBq/kg)
	^238^U	^234^U	^230^Th	^210^Po	^234^U/^238^U	^230^Th/^234^U	^232^Th
Min	0.32 ± 0.09	0.28 ± 0.08	0.1 <	0.8 ± 0.2	0.8 ± 0.2	0.0008 ± 0.0003	0.1 <
Max	1,183 ± 30	1701 ± 43	26 ± 3	94 ± 4	1.97 ± 0.42	0.61 ± 0.31	8.8 ± 2.7
Average	155.8	210.1	2.4	10.5	1.32	0.098	0.9
SD	272.2	375.1	5.0	17.9	0.24	0.157	1.6

Data are given as minimum, maximum and mean values together with SD. Uncertainties are given in brackets.

Sorting out ^238^U activity concentration in pit lake waters, the higher values correspond to sites (mBq/kg):

$${\text{S}}21 \, \left( {1183} \right) \, > {\text{ S}}15 \, \left( {735} \right) \, > {\text{ S}}4 \, \left( {680} \right) \, > {\text{ S}}3 \, \left( {609} \right) \, > {\text{ S}}8 \, \left( {236} \right) \, > {\text{ S}}2 \, \left( {154} \right)$$

Enhanced levels of ^238^U were found and it is well known the potential risk to population due to the chemical and radiological toxicity of ^238^U, with the two important target organs being the kidneys and the lungs.

In this sense, it is worth mentioning that one of the pit lakes is nowadays used as tap water reservoir (Site 2). According to WHO^35^ there is a guidance level of 10 Bq/L in drinking water for ^238^U from the radiotoxic perspective, which is by far one order of magnitude higher than the maximum ^238^U level found in this sampling. However, due to the chemotoxicity of U a more restricted threshold of 30 µg/kg (370 mBq/kg) was defined by WHO in 2011. In our case, the ^238^U activity concentration at Site 2 was 150 mBq/kg which is 2.4 times lower than the threshold, so the chemotoxicity of U is not relevant for local inhabitants.

On the other hand, most of the lakes studied are used for recreational purposes (i.e. fishing, swimming, diving). Although dermal contact is considered a relatively unimportant path of exposure due to the limited transfer from skin to the blood, another possible routes of radionuclide incorporation or impact should be considered from the dose assessment perspective.

**Figure 5:**
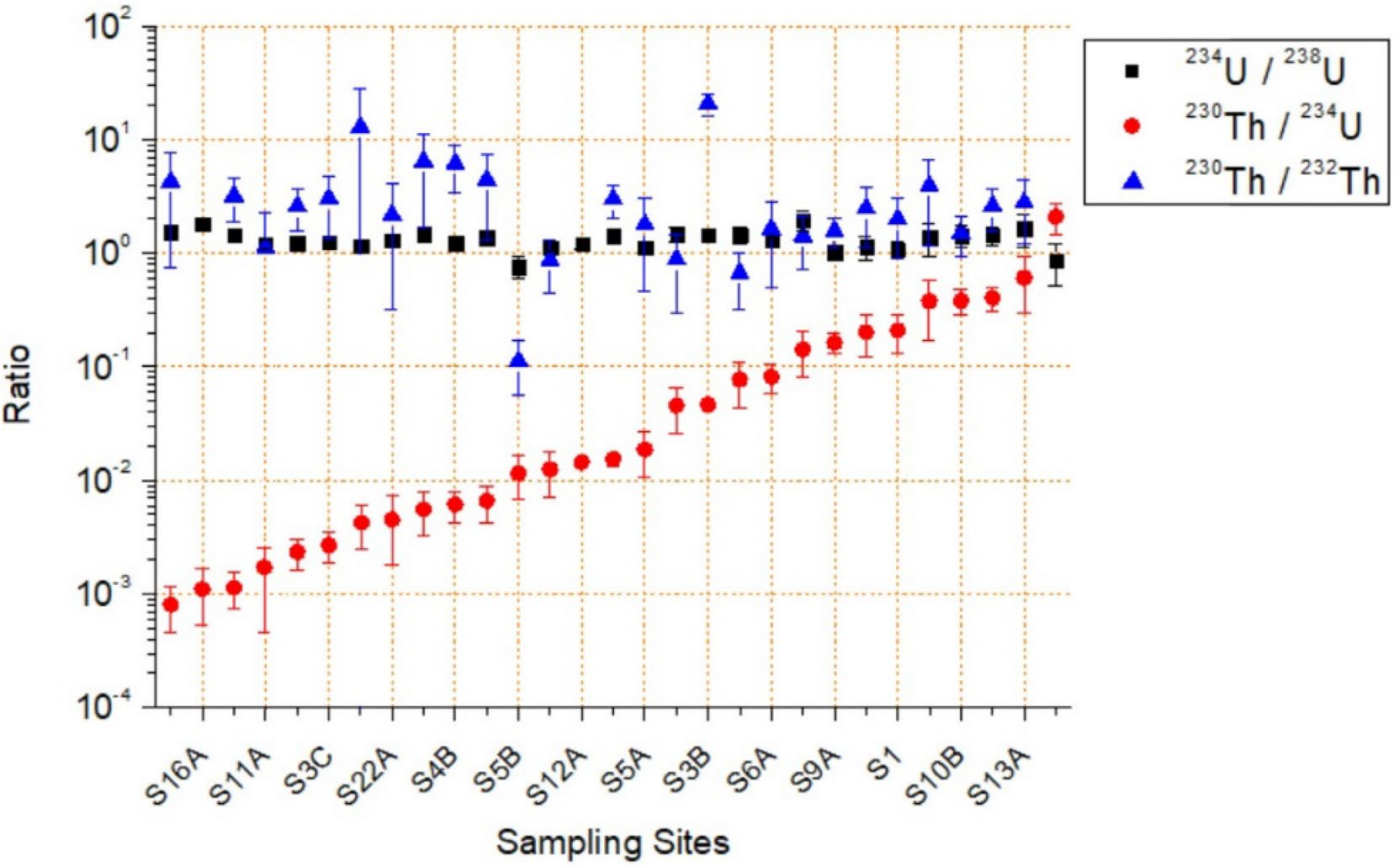
Isotopic ratios showing disequilibrium in ^238^U series for pit lakes water samples. Sampling sites were sorted out attending to an increasing ^230^Th/^234^U ratio. Raw data can be found in Table S4.

Regarding ^234^U, most of the samples had higher values than ^238^U, ranging from 0.3 to 1,700 mBq/kg with a mean of 210 ± 375 mBq/kg. The ^234^U/^238^U activity concentration ratios of pit lake water samples (Fig. 5) were all above unity except for one site. The ^234^U to ^238^U ratio should be 1 in case of secular equilibrium but it is well known that there may exist a disequilibrium in water with ^234^U/^238^U ratios above unity, due to a selective leaching, alpha-recoil transfer of ^234^Th directly into the aqueous phase and the combination of the two processes^36^. The ratio between these radionuclides in surficial water is typically 1.1 to 1.3, with higher values related to a major input of underground water into the water body. The present results are consistent with the expected fractionations based on the greater mobility of U and particularly ^234^U^37^.

##### Thorium radioisotopes in surface water

The activity concentration average of ^230^Th (belonging to ^238^U series) was of 3.1 mBq/kg ranging from MDA (~ 0.1) to 26 ± 3 mBq/kg. The range of values found belongs to the typical environmental values for this isotope. Thorium concentration in water is expected to be very low^38,39^ although it can increase due to soluble complexes with humic material or carbonates^40^. These low values compared with those of ^238^U (and also compared with ^234^U, the ^230^Th direct ancestor) can be explained by a very low mobilization of Th and its tendency to be fixed to the solid phase. As a consequence, ^230^Th/^234^U activity ratios were all found lower than unity (Table 1 and Fig. 5) with a minimum value of 8·10^–4^.

Regarding ^232^Th and its daughters, the activity concentration levels were even lower than ^230^Th (Table 1 and Fig. 5), in accordance with Jia et al.^41^. The MDA for ^232^Th was 0.5 mBq/kg, and 46% of the samples had levels below MDA. ^232^Th activity concentration varied from MDA to 8.8 ± 2.7 mBq/kg with an average of 0.9 ± 1.6 mBq/kg. In continental waters ^232^Th ranges from less than 0.008 to 1.50 mBq/kg with mean 0.10 ± 0.16 mBq/kg^18^. In a direct comparison between Th isotopes, the ratio ^230^Th/^232^Th is mostly higher than 1 (Fig. 5), pointing out the different origin of these two radioisotopes.

Due to the low activity concentration of Th isotopes, these radionuclides will have a negligible radiological impact on exposed individuals.

##### ^210^Po in surface water

Average activity concentration of ^210^Po, also belonging to ^238^U series, in surface water samples was 10.5 ± 17.9 mBq/kg with a variation from 0.8 to 95 ± 4 mBq/kg. The distribution of these data can be seen in Fig. 4b where 94% of the samples had values below 25 mBq/kg and 76% below 10 mBq, which is in agreement with environmental levels^42^.

From a dosimetric perspective, ^210^Po is the radionuclide with the highest ingestion dose coefficient what implies the higher radiological toxicity. As an example, in Site 2 (mentioned before) a pit lake used as water reservoir for human consumption, a straightforward assessment of the annual committed effective dose via ingestion is showed in Table 2. From this table, the multiplication of activity concentration, annual intake of water (assumed to be 2 kg/day in adults) and the effective dose coefficient by ingestion for adults, provides the annual dose by ingestión due to water including ^238^U, ^234^U and ^210^Po radionuclides (Th isotopes are neglected for being below MDA). Taking into account that the threshold for the effective dose in water is 0.1 mSv/y^43^, the total amount (0.0136 mSv/y) represents only 14% of this threshold.

**Table 2:** Annual committed effective doses (mSv/y) by ingestion in adults due to water consumption from pit lake site 2.

Radionuclide	Activity concentration in water (Bq/kg)	Water consumption in adults per year (kg/year)	Committed effective dose coefficient by ingestion (Sv/Bq)^a^	Annual dose by ingestion (mSv/year)
^238^U	0.154	730	4.5·10^–8^	0.0051
^234^U	0.164	730	4.9·10^–8^	0.0059
^210^Po	0.003	730	1.2·10^–6^	0.0026
			Total	0.0136

^a^From ICRP, 2012^51^.

### Elemental and radiometrical characterization of sediments

#### Elementary characterization

**Figure 6:**
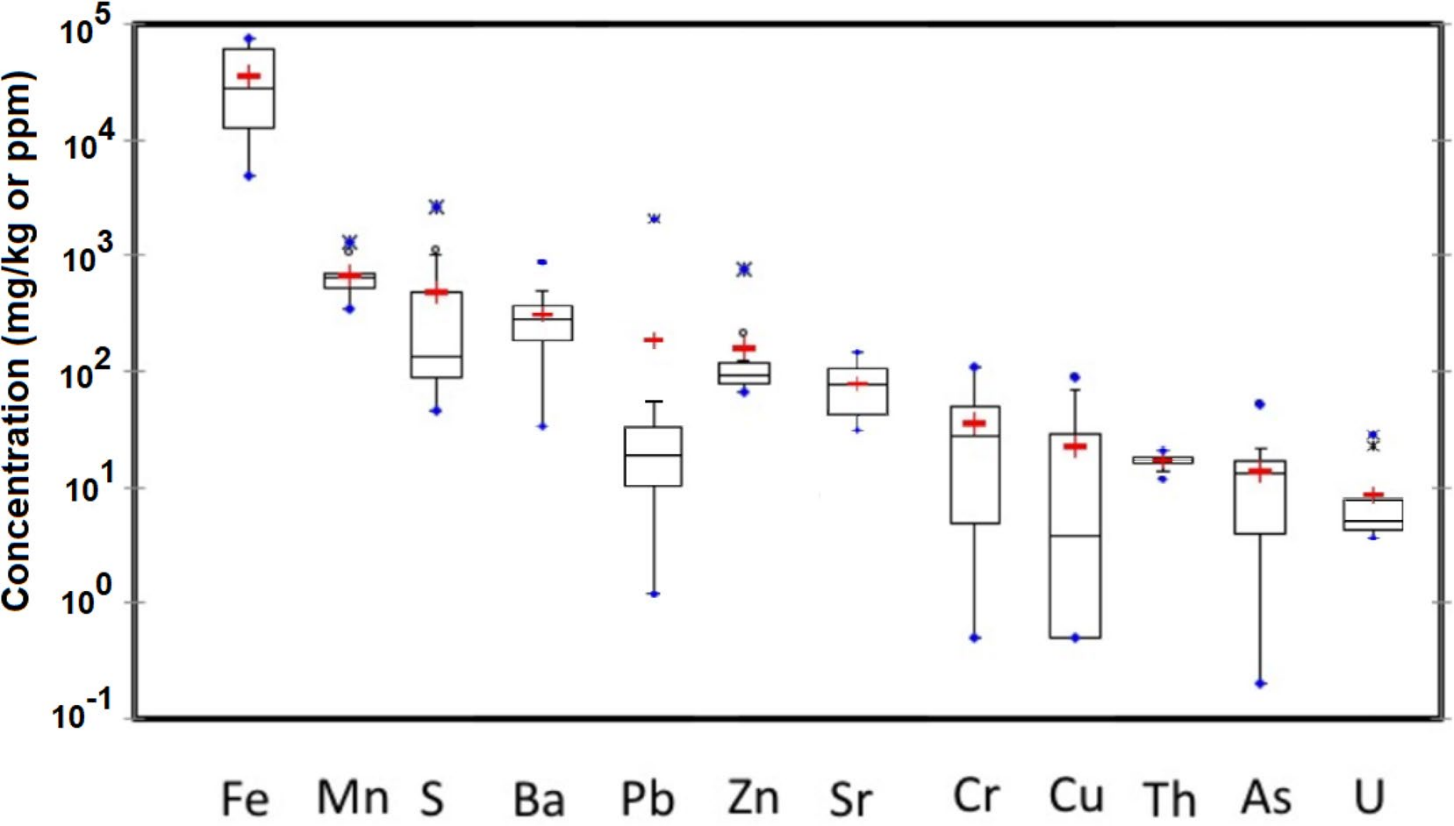
Concentration of major and trace metals in sediments (n = 14) from pit lakes, presented as box-and-whisker plots and sorted out by mean values. For explanation of the boxes and whiskers, refer to Fig. 2.

The abundance of major and trace elements was determined in 14 sediment samples by XRF (Fig. 6), and the observed composition reflects accurately the lithological characteristics of the study area. Raw data to produce Fig. 6 can be found as Table S5 in supplementary material. The most abundant element is Si (62% of average), followed by Al (9.8%) present in aluminosilicate. The presence of Ca (average of 4.9%) suggests the influence of liming in the studied lakes, although the existence of derelict marble quarries among the sampling sites could also explain such values. Lower abundance of K, Mg, and Na was observed (3.2, 3.0 and 1.0%, respectively) related to the weathering of bedrock. The presence of oxides and hydroxides seems to be limited to Fe (average value of 4.8%), considering the low concentration of Mn in sediments (0.1%).

Concerning trace metals, a crustal element like Ba was among the most abundant in sediments (309 ppm), followed by Pb (285 ppm), Zn (163 ppm), Sr (82 ppm), Cr (45 ppm), Cu (41 ppm), Th (18 ppm), As and Ni (14 ppm) or U (12 ppm). Liming of lake waters may have caused a net transference of metals from the water column to the lake sediments due to increase of pH values. In order to estimate the metal fluxes from the water column to the sediment and vice versa, assessment of distribution coefficients (K_d_) have been performed.

**Figure 7:**
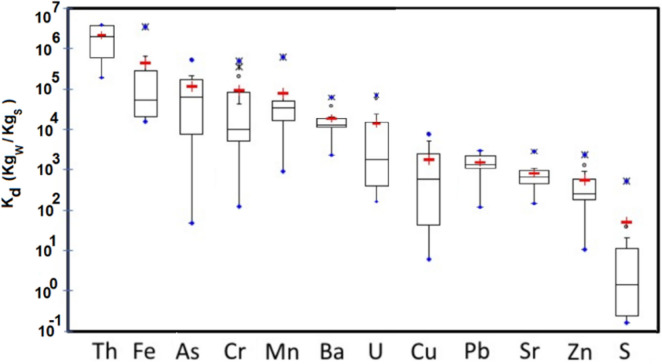
Distribution coefficient (K_d_) for elements in pit lakes (n = 14) and sorted out by mean values. For explanation of the boxes and whiskers refer to Fig. 2.

Distribution coefficient (K_d_) was estimated as the ratio between sediment and water concentration for an element. In some water and sediment samples, values below the detection limit were observed for some elements, and in such cases half of the detection limit was assumed in order to be considered in this K_d_ analysis. Some clear trends can be observed (Fig. 7): S (associated with sulfides) with low K_d_ can easily move to the aqueous phase, while the opposite behavior was found for Th with the highest K_d_ showing a clear tendency to remain in the solid phase. Intermediate values were obtained for the other trace elements having U with ranges of K_d_ values similar to Cr, Cu, Zn, As, Sr, Ba or Pb. Based on K_d_ average values and interquartile ranges, we can sort out the trend to be mobilized into the aqueous solution for the elements in pit lakes as follows:

$$\left( {{\text{Highest mobility}},{\text{ i}}.{\text{e}}.{\text{ lowest K}}_{{\text{d}}} } \right){\text{ S }} > {\text{ Cu }}\sim {\text{ Zn}}\sim {\text{ P }} \ge {\text{ U }} \ge {\text{ As}}\sim {\text{ Cr}}\sim {\text{ Ba }} > {\text{ Fe }} > {\text{ Th }}\left( {\text{Lowest mobility}} \right)$$

Nguyen et al.^44^ reported K_d_ values for various natural aquatic systems (Australian coastal and estuaries, six estuaries in Texas and in several other natural lakes), with log K_d_ ranges of 3.0–5.9, 3.8–6.7 and 3.8–7.2 for Cu, Zn and Pb, respectively. In our survey of pit lakes, we found log K_d_ ranges of 0.78–3.9, 1.0–3.4 and 2.1–3.5 for Cu, Zn and Pb, respectively. After comparison of these data sets, it is clear that the mobilization of these metals into the aqueous phase in the surveyed pit lakes is higher than in natural water environments (lower K_d_ values), once again demonstrating the need to study these special water bodies for the potential risk as a source of heavy metals in the surrounding environment.

As a tool to identify whether there exist or not a chemical pollution risk to the environment in a pit lake, sediment elementary composition can be used according to Håkanson^29^ proposal. In this model, after aplying Eq. (1), both C_f_ and C_d_ are classified in 4 levels: low, moderate, considerable and very high (Table 3). In the present work, Hg and PCB concentrations were not determined and Cd was found below detection limit (0.1 ppm) in all sediments. Thus 6 metals were included in the assessment what implies a conservative C_d_ value. Attending to calculated values (Table 3), only one site (Site 14) was found with a *very high C*_*d*_ value, mainly due to a *very high* Pb C_f_, together with *considerable* C_f_ values of Zn and As. This lake is a well-known site for local people due to its “turquoise” water and commonly used for diving and swimming (see supplementary material file, picture 23).

**Table 3:**
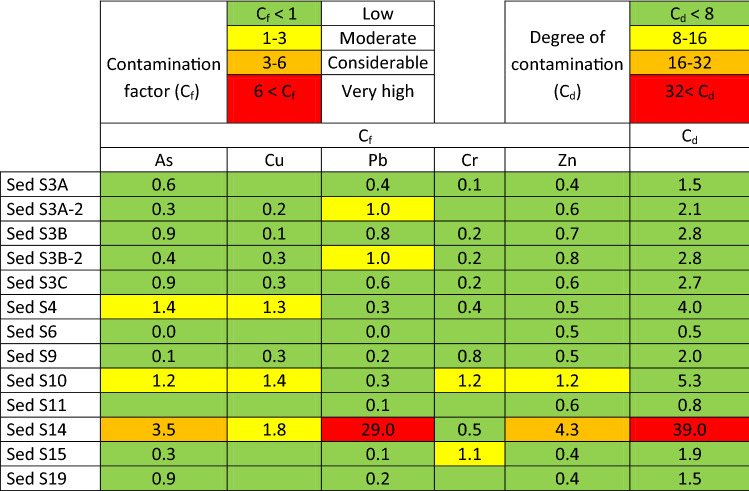
Contamination factor (C_f_) and degree of contamination (C_d_) in sediments from pit lakes for selected elements.

The results are colour coded according to the levels proposed by Håkanson (1980).

#### Radiometrical characterization of sediments

In this section the concentration of NORM radionuclides, determined by means of alpha and gamma spectrometry in a set of 14 sediment samples is reported. U, Th and Po activity concentrations of radionuclides from ^238^U and ^232^Th series, determined by alpha spectrometry are shown in Table 4 (raw data to produce this table can be found as Table S6 in supplementary material).

**Table 4:** U, Th and Po activity concentrations of radionuclides from ^238^U series, ^235^U and ^232^Th in pit lake sediments (n = 12) determined by alpha spectrometry.

(Bq/kg)	^238^U	^234^U	^230^Th	^232^Th	^210^Po	^235^U
Min	4.7 ± 0.4	4.2 ± 0.4	13 ± 2	11 ± 2	15 ± 1	0.05 ± 0.03
Max	1,190 ± 35	1,390 ± 40	1,400 ± 86	112 ± 9	1,690 ± 110	48 ± 3
Average	217	232	223	62	257	7.8
SD	384	448	432	54	576	15.2

Values are given as range (minimum and maximum), average and standard deviation.

These alpha spectrometry data were based on total digestion of the samples, and it should be noted that differences in activity concentration of U, Th and Po isotopes will depend on the digestion method used^25^. In that work the ratio was in general higher than unity (demonstrated in a subset of 10 sediment samples from Swedish pit lakes), and it was concluded that Th isotopes are more bound to the “undissolved fraction” than U and Po isotopes, which is in good agreement with the results presented above.

Gamma spectrometry results are presented in Table 5. Regarding the ^238^U series: ^234^Th, ^226^Ra (via ^214^Bi and ^214^Pb) and ^210^Pb, the activity concentrations agreed within 2-sigma criteria in most of the cases. Hence, we can conclude that there is practically secular equilibrium in sediments for the ^238^U series radionuclides (with only ^210^Pb in some cases having some excess, unsupported ^210^Pb). In connection with alpha measurements (Table S6 in supplementary material), ^238^U and ^234^U (via alpha) fit with ^234^Th (via gamma)^25^ and ^210^Po (via alpha) matches with ^210^Pb what proves the ^210^Pb-^210^Po secular equilibrium in the lower part of the ^238^U series in this set of sediments. It should be noted that the measured sediments originate from the shoreline and not from the deepest part of the lake. Additionally, due to the enhanced levels of ^238^U for some sediment samples values of ^235^U above gamma spectrometry MDA were found as well in 6 samples. In these samples the average ^238^U/^235^U ratio was 20.2 ± 4.0, which is in good agreement with the natural ratio ^238^U/^235^U of 21.4 in the environment^36^. Concerning alpha spectrometry, due to a lower MDA, ^235^U was measured in 11 samples and the average ^238^U/^235^U ratio was 22.4 ± 3.1, same accuracy but a more precise result than the obtained by gamma spectrometry.

**Table 5:** Radionuclide activity concentration in sediments (Bq/kg) based on gamma spectrometry measurements.

Sediment site number	^238^U series			^232^Th series	^235^U series	^137^Cs	^40^K
	^234^Th	^226^Ra	^210^Pb				
S3A	336 ± 11	347 ± 12	299 ± 5	47 ± 2	19 ± 2	< 0.8	1,354 ± 44
S3A-2	381 ± 42	371 ± 15	360 ± 14	72 ± 5	26 ± 6	< 1.8	1,210 ± 74
S3B	1,472 ± 57	1,301 ± 50	1,353 ± 44	172 ± 17	79 ± 9	5.3 ± 0.9	1,405 ± 52
S3B-2	1,366 ± 42	1,251 ± 45	1,379 ± 28	173 ± 10	63 ± 6	5.9 ± 0.8	1,363 ± 60
S3C-2	828 ± 21	1,003 ± 99	964 ± 10	136 ± 6	42 ± 7	1.3 ± 0.2	972 ± 38
S4A	77 ± 5	96 ± 5	82 ± 4	68 ± 4	< 6.6	< 0.7	971 ± 32
S6A	8 ± 2	10 ± 1	14 ± 2	7 ± 2	< 7.2	< 0.8	56 ± 9
S9A	58 ± 9	63 ± 4	92 ± 7	35 ± 1	< 7.8	0.9 ± 2	734 ± 27
S10A	33 ± 3	44 ± 2	47 ± 3	32 ± 2	< 7.9	2.5 ± 4	499 ± 22
S11B	17 ± 3	18 ± 1	20 ± 1	15 ± 2	< 8.2	< 0.5	382 ± 15
S14A	21 ± 2	31 ± 3	51 ± 3	31 ± 3	< 6.7	5.8 ± 7	724 ± 26
S15A	73 ± 9	87 ± 3	85 ± 3	69 ± 4	< 7.1	0.6 ± 1	738 ± 27
S19A	20 ± 3	26 ± 2	28 ± 3	18 ± 3	< 8.7	2.7 ± 4	437 ± 21
S21A	1,070 ± 35	1,420 ± 30	715 ± 12	538 ± 37	49 ± 6	1.7 ± 5	1,162 ± 53
S22A	238 ± 17	263 ± 5	217 ± 20	162 ± 15	12 ± 4	< 1.0	1,418 ± 49
S23A	514 ± 7	258 ± 33	520 ± 15	246 ± 13	20 ± 2	1.8 ± 2	1,138 ± 38

^232^Th results comprise the average of ^228^Ac, ^212^Pb, ^212^Bi and ^208^Tl.

A clear secular equilibrium was found for radionuclides in the ^232^Th series (^228^Ac, ^212^Pb, ^212^Bi and ^208^Tl), matching all radionuclides within k = 1 criteria uncertainty. For that reason, the reported value of ^232^Th-series refers only to ^232^Th. These values, as well as the corresponding values for ^137^Cs and ^40^K activity concentrations are shown in Table 5.

According to UNSCEAR^45^ the natural radionuclide content for soils in Sweden range (in Bq/kg): from 12–170, from 14–94 and from 560–1,150 for ^226^Ra, ^232^Th and ^40^K, respectively. The values obtained from the sediment samples are to a high extent within these ranges (Tables 4 and 5), except for sites S3, S21, S22 and S23, where isotopes from U, Th series and ^40^K (in some cases) levels exceed these values. It should then be noted that sites S3 and S21 also belong to those with the highest ^238^U activity concentrations in water samples.

#### Radionuclide K_d_ values

In order to assess the K_d_, defined as the ratio between the activity concentration in sediment and the one in water, the data for U, Th and Po isotopes should in general be based on alpha spectrometry of sediments applied to total digestion and of water samples. However, to save time and resources, gamma spectrometry can be used for sediment samples when secular equilibrium in a decay series occurs^25^. Secular equilibrium between ^238^ and ^234^U was confirmed in sediments via alpha spectrometry, so ^234^Th may be analyzed instead. Furthermore, a comparison between ^232^Th via alpha and ^228^Ac via gamma spectrometry, confirmed a secular equilibrium, and hence the latter radionuclide can represent the activity concentration of the parent of the series. Regarding ^230^Th in sediments, ^226^Ra-^234^Th equilibrium was confirmed (with ^230^Th in between). Additionally, ^210^Po would require ^210^Pb-^210^Po equilibrium (to some extent also achieved within this set of sediments), so we can use ^210^Pb instead. If all these equilibria are fulfilled, alpha spectrometry in water and gamma spectrometry in sediment could then be used to obtain appropriate K_d_ values.

**Figure 8:**
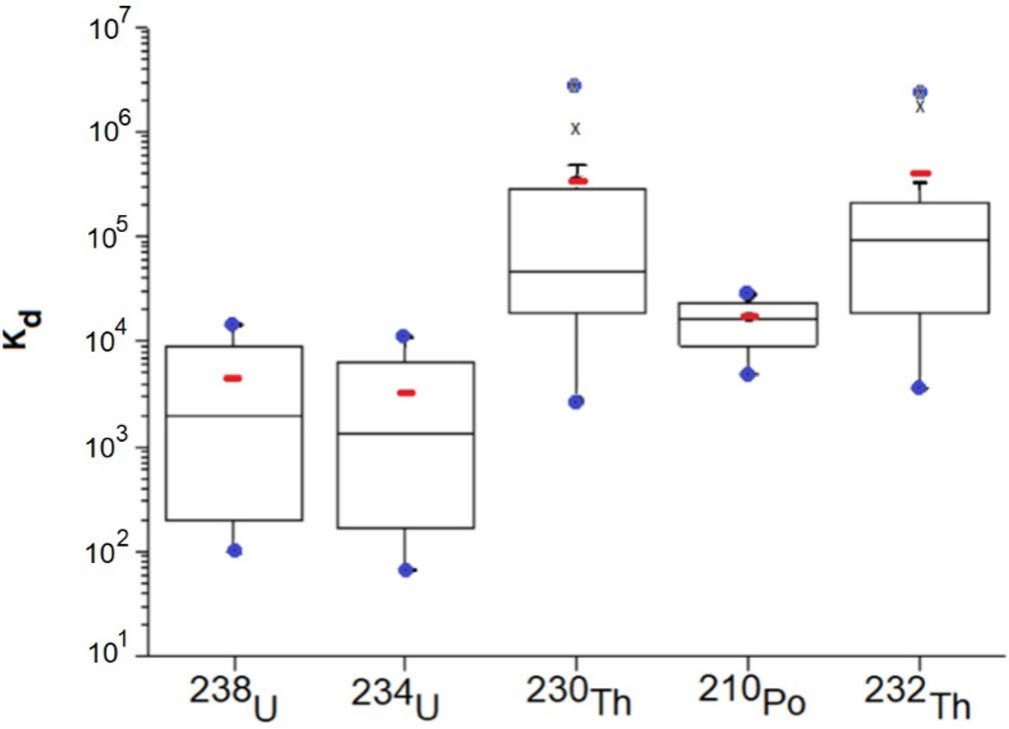
Distribution coefficient (K_d_) for radionuclides (n = 14) for the studied pit lakes. Sediment samples were measured by gamma and water samples by alpha spectrometry, assuming secular equilibrium. For explanation of the boxes and whiskers refer to Fig. 2.

Using the described methodology, K_d_ values were assessed for each radionuclide (Table 6 and Fig. 8). Raw data can be found in Table S7, supplementary material. It can be observed that ^238^U and ^234^U have a similar behavior although ^234^U has a slight higher tendency (k_d_ average 3,500) than ^238^U (k_d_ average 4,700) to mobilize into the aqueous phase, confirming what was obtained through disequilibrium analyses of ^238^U series in water. And the same applies to ^230^Th and ^232^Th, i.e. similar behavior between isotopes although ^230^Th (k_d_ average 2.9·10^5^) has a higher trend to incorporate into the aqueous solution in comparison with ^232^Th (k_d_ average 3.9·10^5^). This behaviour can likely be connected with the alpha-recoil transfer of ^234^U directly into the aqueous phase, producing this preference to leach for ^230^Th compared with ^232^Th. Additionally, K_d_ ranges were in good agreement for U and Th isotopes (Fig. 8) and the ones for elemental U and Th (Fig. 7), implying a good performance between the 4 different techniques involved in this assessment (ICP-MS, XRF, alpha and gamma spectrometry). Concerning ^210^Po isotopes, a higher K_d_ was observed than for U isotopes (impliying lower fractionation into aqueous phase than U), but lower K_d_ than Th isotopes, implying a higher fractionation into water than Th.

**Table 6:** Distribution coefficient K_d_ for radionuclides in pit lakes (n = 14).

K_d_	^238^U	^234^U	^230^Th	^210^Po	^232^Th
Min	9.9E+01	6.8E+01	2.3E+03	2.3E+03	3.7E+03
Max	1.4E+04	1.1E+04	2.2E+06	6.2E+04	2.4E+06
Average	4.7E+03	3.5E+03	2.9E+05	2.3E+04	3.9E+05
SD	5.1E+03	3.7E+03	5.9E+05	1.7E+04	7.4E+05

Values are given as range (minimum and maximum), average and standard deviation.

The aforementioned results can be summarized by ranging the radionuclides mobility into the aqueus solution in pit lake waters in the following way:

$$\left( {{\text{Highest mobility}},{\text{ i}}.{\text{e}}.{\text{ lowest K}}_{{\text{d}}} } \right)^{{{234}}} {\text{U }} >^{{{238}}} {\text{U }} >^{{{21}0}} {\text{Po }} >^{{{23}0}} {\text{Th }} >^{{{232}}} {\text{Th }}\left( {\text{Lowest mobility}} \right)$$

### Ambient dose rate equivalent

**Figure 9:**
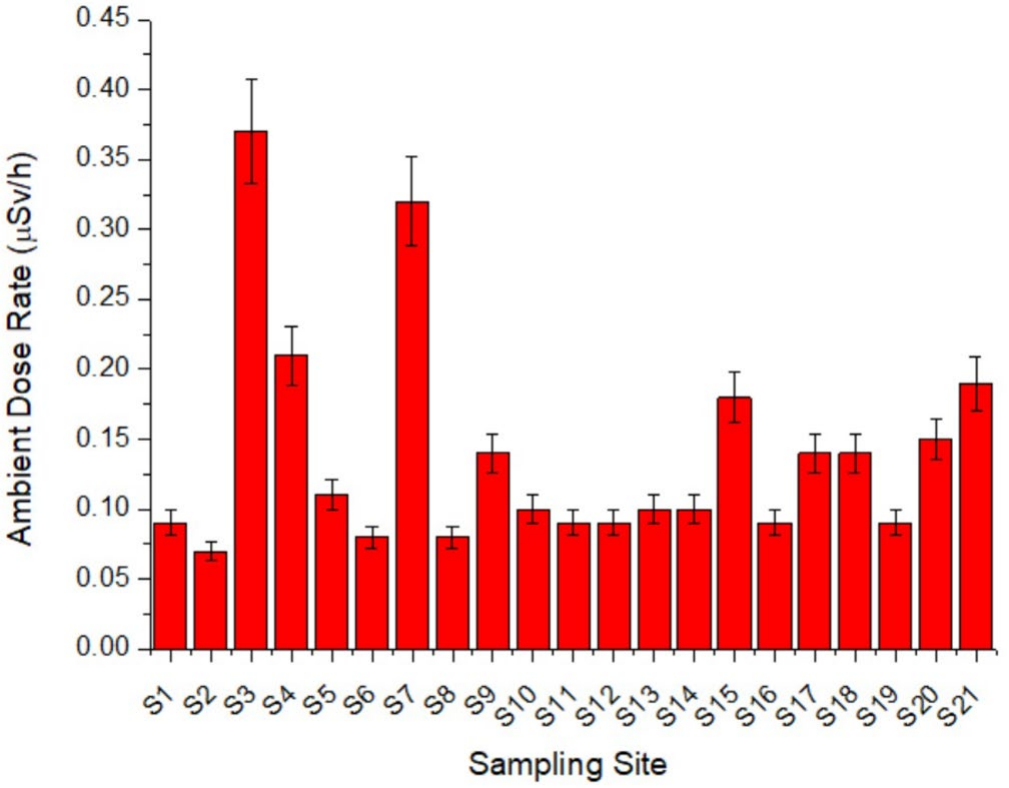
Ambient dose rate equivalent (μSv h^−1^) in pit lakes from southern Sweden.

In Fig. 9, the ambient dose rate equivalent is plotted in terms of ambient dose rate. The values range from 0.06 to 0.37 μSv h^−1^ with an average value of 0.14 ± 0.08 µSv h^−1^ that is a typical environmental value. Note that Sites 22 and 23 were not measured due to technical problems during the sampling campaign. Thus, several sites with enhanced ambient dose rate levels compared with environmental average values (0.14 μSv h^−1^) were identified, with the five sites with highest values (in μSv h^−1^):

$${\text{S3 }}\left( {0.{37}} \right) \, > {\text{ S7 }}\left( {0.{32}} \right) \, > {\text{ S4 }}\left( {0.{21}} \right) \, > {\text{ S21 }}\left( {0.{19}} \right) \, > {\text{ S15 }}\left( {0.{18}} \right)$$

### Rock samples

**Figure 10:**
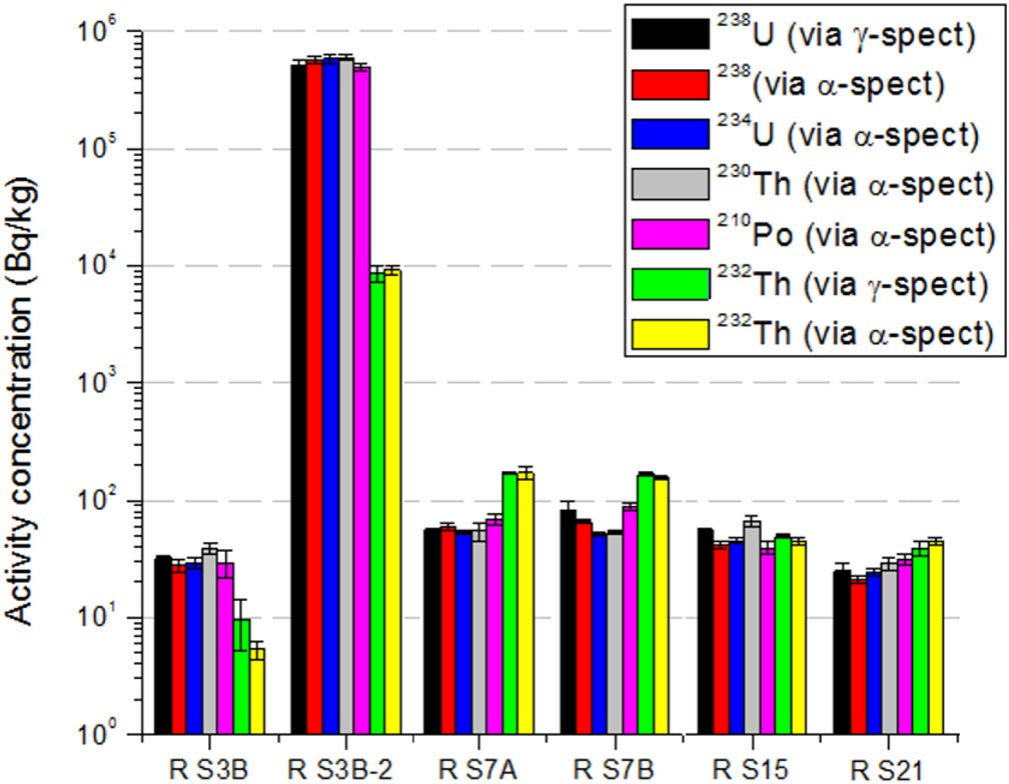
Activity concentration of selected radionuclides in rock samples based on gamma and alpha spectrometry from four of the five pit lake sites with highest external gamma dose rates.

Rock samples were collected and measured by gamma and alpha spectrometry (using total digestion) from four out of the five sites with highest ambient dose rate equivalent (except site 4). Secular equilibrium was found in all cases within 1-k criteria uncertainty for activity concentrations of ^238^U, ^235^U and ^232^Th natural series (Fig. 10), demonstrating good agreement between gamma and alpha spectrometry results. Concerning ^238^U to ^232^Th series ratios, ^238^U series activity concentration was higher than that of the ^232^Th series at site S3, while at sites S7 and S21 the opposite was found. Site S15 shows this ratio compatible with unity.

Large heterogeneities regarding activity concentration of radionuclides were found in rocks from a single site, and a clear example occurs at site 3B (labelled as RS3B and RS3B-2). The RS3B-2 sample (from a former feldspar mine) exhibit activity concentrations of 513 ± 50, 22.6 ± 1.5 and 8.7 ± 1.4 Bq/g for ^238^U, ^235^U and ^232^Th, respectively, indicating an approximate 4% mass content of U. According to IAEA^46^, any material exceeding 1 Bq/g of ^238^U or ^232^Th will require further investigations in terms of its use or storage. The radiological assessment becomes a relevant study to address because this site is quite often visited by people for recreation. The activity ratio ^238^U/^235^U of 22.7 ± 2.7 is in good agreement with the natural ratio 21.4.

A detailed examination by SEM–EDX was applied to two rocks from sites S3B and S7, with the highest ambient dose rate equivalents, to determine the morphology and chemical composition of minerals forming the bedrock, which could explain the high dose rates found.

#### SEM–EDX for rocks from site S7

**Figure 11:**
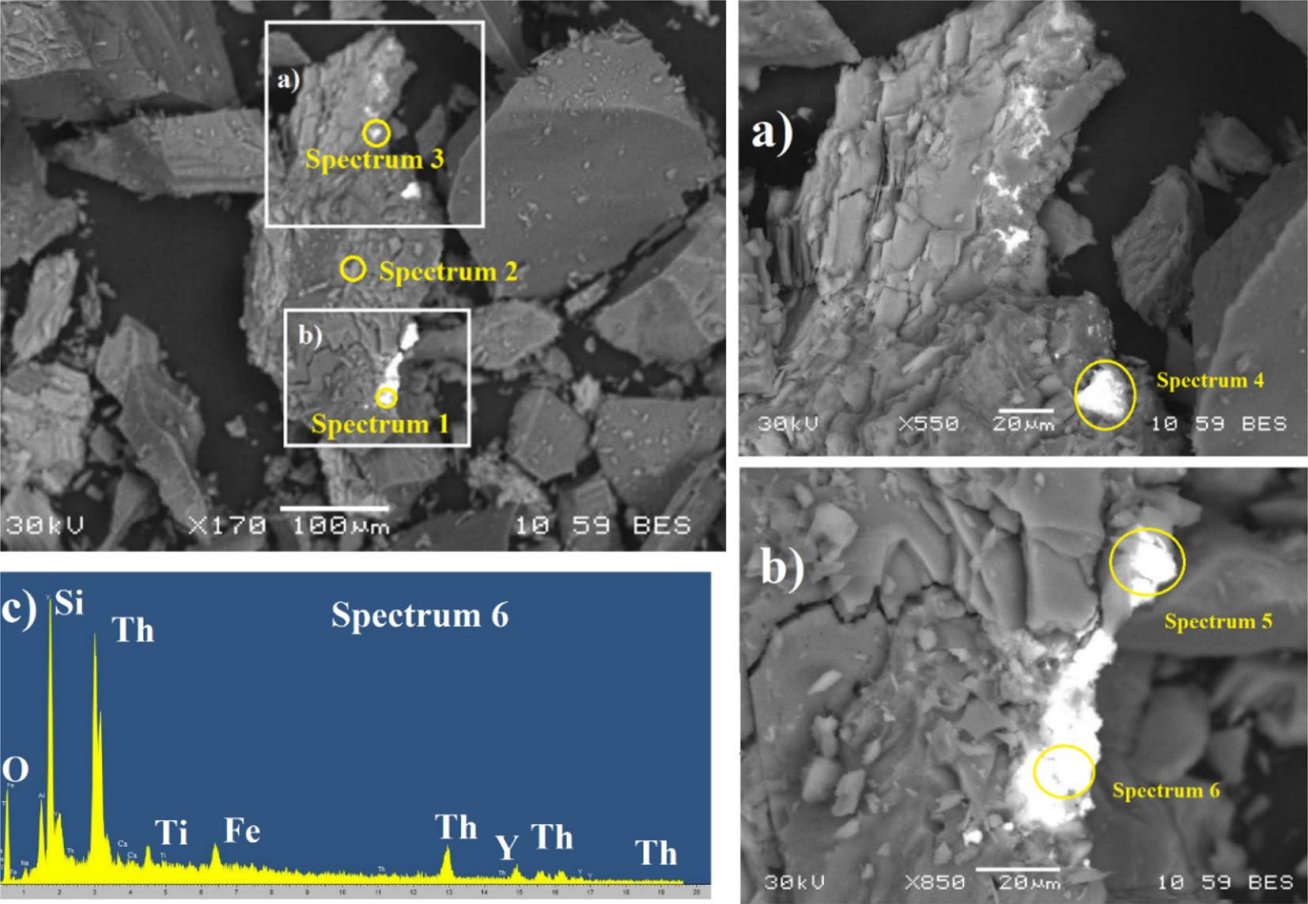
(**a**) and (**b**) SEM Backscattered images of rock sample RS7 marking spots where six different spectra were recorded. c) SEM–EDX spectra from spot 6 (spectrum 6).

The bedrock outcropping in the surrounding of Site 7 is mainly composed of granite and pegmatite, formed by quartz, feldspar and mica, although other minor minerals can be found. Figure 11a,b shows SEM backscattered images (BES) and combined with the data presented in Table 7, a predominance of particles of aluminosilicate and Fe oxides can be detected. However, the presence of brighter particles denoting the presence of elements of higher atomic number is also observed. Semi-quantitative analyses on this sample show high concentrations of Y (up to 11%) and Th (up to 28%) according to Fig. 11c and Table 7. The presence of these elements may be related to the accessory mineral assemblage commonly found in granites, such as monazite, xenotime, Th-ortosilicate and uraninite^47^. The weathering of these minerals may lead to the release into the lake sediments although due to the topography of site S7, no sediments could be collected at this site.

**Table 7:** Semi-quantitative elementary composition (% weight) at 6 spots acquired from RS7.

(% weight )	C	O	Na	Al	Si	P	K	Ca	Ti	Fe	Y	Zr	Th	U
Spectrum 1	5.2	43		1.9	11	1.5		0.4	1.2	1.6	6.3		28	
Spectrum 2		41		14	24		12			8.9				
Spectrum 3	14	48	0.7	2.3	17		0.8		0.5	3.9	6.7		3.9	2.3
Spectrum 4	8.2	42		1.3	17	0.5			1	3	11	0.8	10	5.1
Spectrum 5	5.7	50		2.4	11			0.4	0.9	1.5	4.3		25	
Spectrum 6	4.3	33	1.1	3.3	13			0.7	1.5	2	8.6		32	

#### SEM–EDX for rocks from site S3

**Figure 12:**
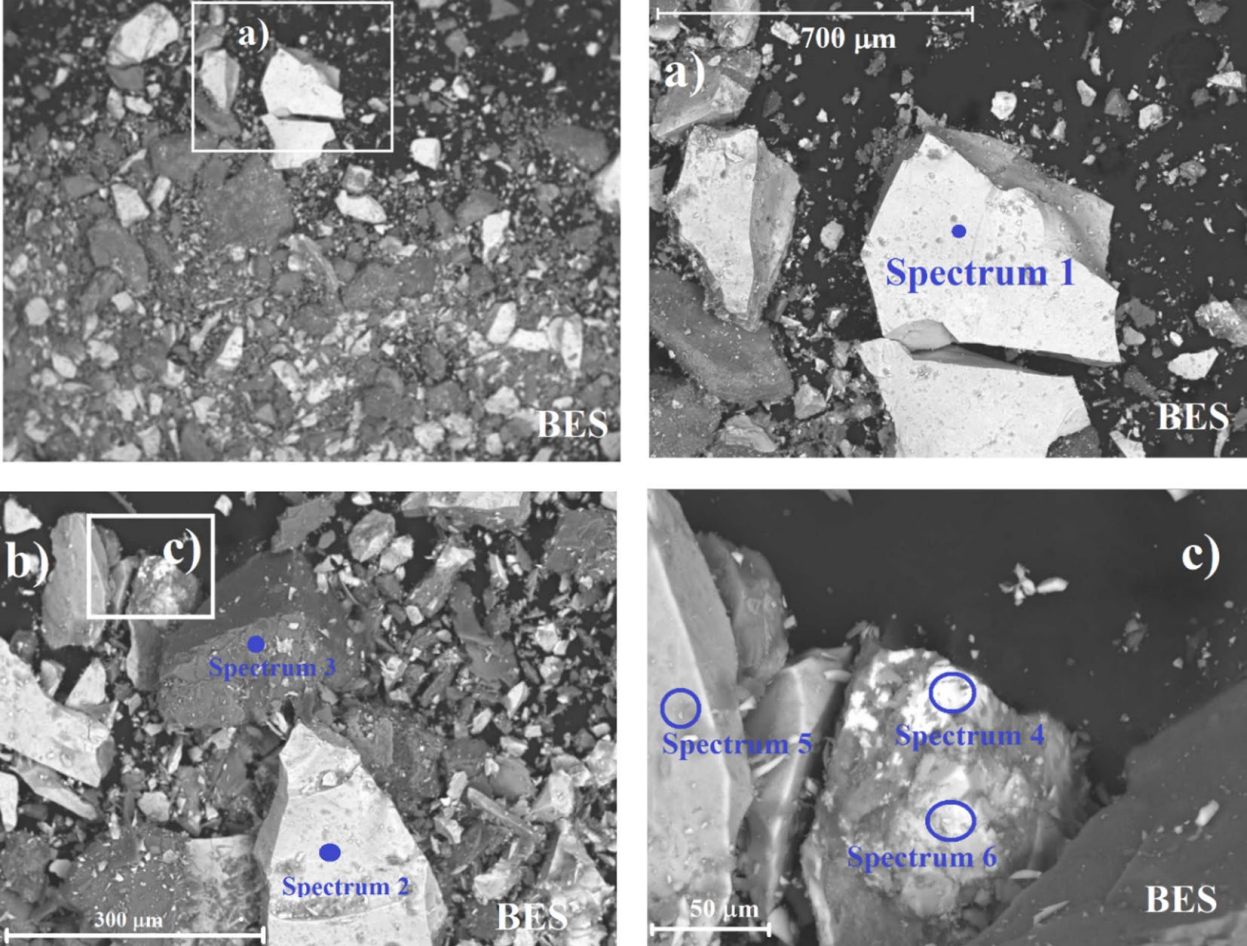
SEM Backscattered images of rock sample RS3B-2, and spots where the spectra were taken.

Different materials compose the bedrock surrounding site 3. The predominant rocks in the drainage area are granitoids and syenitoids, although the presence of volcanic acid rocks such as rhyolite and dacites are also observed. Figures 12a–c shows backscattered images of rock samples collected at site 3. The results from Table 8 show presence of aluminosilicates and Fe oxides, as well as tantalum and niobium. Ta and Nb mineralization is often associated with geochemically specialized granites which are characterized by enrichment in fluorine, and by the development of pervasive, postmagmatic alteration^48^. Similar to the case of site 7, the occurrence of accessory minerals (e.g. zircon, Th-orthosilicate and uraninite) in granite may explain the high concentrations of Th (up to 32%) and U (up to 8.9%) observed in this area. It is worth to point out the huge size of the heaviest particles in this sample (Fig. 12a). The presence of U and Th-enriched zircon as accessory mineral of pegmatites (similar composition as granites) caused enhanced levels of radionuclides in ground waters of SW Niger^49^. Therefore, water interacting with such materials need to be studied in the long term.

**Table 8:** Semi-quantitative elementary composition (% weight) at 6 spots acquired from RS3B-2.

(% weight)	C	O	Al	Si	K	Ca	Ti	Fe	Mn	Y	Nb	Yb	Ta	W	Th	U
Spectrum 1		37.4		2.2		3.5	0.4	8.9		7.7	23.6	1.1	3.0	5.0		7.1
Spectrum 2		39.0	1.2	5.3		4.4	0.3	10.8		2.3	22.8		3.2	1.8		8.9
Spectrum 3		18.4	9.1	14.9	2.4	0.7		45.5	5.0		3.3					
Spectrum 4	2.5	34.1		7.8		1.4		6.5		4.7	5.4	1.5			32.5	3.6
Spectrum 5	7.9	46.8	1.4	3.7		3.5	0.3	5.9		3.1	18.0		1.6	2.3		5.7
Spectrum 6		35.7		6.6		2.1	0.3	5.8		5.0	10.7	1.3			29.0	2.3

## Conclusions

Radiometric and metal levels of 23 mine sites containing former mining pit lakes in southern Sweden were examined. The survey was focused on water, sediment and some rocks attending to radiometrical and elementary characterization. Additionally, in situ ambient dose rate equivalent was measured. The combination of elemental techniques such as ICP-MS and XRF with alpha and gamma spectrometry were demonstrated to be very useful tools to evaluate environmental levels in former mining areas of Sweden.

From the radiometric point-of-view, enhanced U levels were found in 26% of water samples, whereas Po and Th activity concentrations were found to be at background environmental levels. The highest levels of uranium in surface water were found in an iron and granite mine, so an assumption on radionuclide concentration should not be made firstly on what kind of mine it was, but rather on the bedrock concentration as this will have a much higher impact on the consequent concentrations in the water body. Based on the radionuclides K_d_ values, it was confirmed that the isotopic fractionation for NORM radionuclides in the aquatic environment of pit lakes in southern Sweden can be sorted out in the following way: (higher mobility) ^234^U > ^238^U > ^210^Po > ^230^Th > ^232^Th (lower mobility).

Concerning the radiological perspective, several sites were identified as having moderately elevated ambient dose rate equivalent levels, and rocks from one of these sites were found with up to 4% of U content. So it is a relevant question to have (or generate) maps with dose rates or radionuclide concentrations in the ground as this information is a valuable tool in locating non uraniferous mines with potential enhanced levels of radionuclides. Apart from the environmental risk, the fact that most of these sites are nowadays used by local population for recreational purposes (fishing, swimming or diving) makes the present type of survey an essential tool to identify potential risk to the environment and public health.

Regarding the elementary perspective, higher metal levels were observed in pit lake water compared with that in natural lakes. The metal enrichment in studied sediments were low to moderate in most lakes, with one clear exception where considerable metal enrichment for Cd, Pb and Zn was found. The mobility (the higher mobility, the lower K_d_) of elements into the aquatic media follows this pattern: (higher mobility) S > Cu ~ Zn ~ P ≥ U ≥ As ~ Cr ~ Ba > Fe > Th (lower mobility). A comparison of K_d_ values in pit lakes (from this work) with natural lakes worldwide evidences a higher metal release from pit lakes than from natural lakes.

As main conclusion of this work, it has been proved that measurement of natural radioactivity represents another perspective that should be added in routine analysis of characterization in mining areas worldwide, especially if restauration of post-mining sites is intended for human recreational sites.

## Supplementary information

Supplementary Information 1.
