# Supplementary material for: Pit lakes from Southern Sweden: natural radioactivity and elementary characterization
Source: Sci Rep. 2020 Aug 13;10:13712. doi: 10.1038/s41598-020-70521-0 (PMC7426949; doi:10.1038/s41598-020-70521-0)
Supplement: Supplementary file 1 — Supplementary Information 1. [file 41598_2020_70521_MOESM1_ESM.docx]

**Supplementary material**

**Pit lakes from Southern Sweden: natural radioactivity and elementary characterization**

*J. Mantero ^1,2*^, R. Thomas^1^, E. Holm^1^, C. Rääf ^3^, I. Vioque^2^, C. Ruiz-Canovas ^4^, R. García-Tenorio^2,5^, E. Forssell-Aronsson^1^and M. Isaksson^1^.*

*^1^ Department of Radiation Physics, Institute of Clinical Sciences, Sahlgrenska Academy at University of Gothenburg, Gothenburg,* *SE-413 45 Sweden*

*^2^ Department of Applied Physics II, ETSA, University of Seville, Seville, 41012 Spain*

*^3^ Medical Radiation Physics, Department of Translational Medicine ITM, Lund University, Malmö, Sweden.*

*^4^* *Department of Earth Sciences & Research Center on Natural Resources, Health and the Environment. University of Huelva, Huelva, 21071, Spain.*

*^5^Spanish National Accelerator Centre (CNA), University of Seville, 41092, Spain*

(*)Corresponding author, E-mail: juan.mantero.cabrera@gu.se

This document contains raw data used to produce Figures in the manuscript.

**Table S1.** Coordinates, size of the pit lake, working time and main ores mined in the selected sites. **Page S2**

**Table S2**. Physico-Chemical parameters in Pit lakes water samples from Southern Sweden. **Page S3**

**Table S3**. Elemental composition (mayor and traces) by ICP-MS in Pit lakes water samples from Southern Sweden. **Pages S4-S5**

**Table S4**. U, Th, Po isotopes by alpha spectrometry in Pit lakes water samples from Southern Sweden. **Page S6**

**Table S5.** XRF elemental composition in Pit lakes sediment samples from Southern Sweden. **Page S7**

**Table S6.** Activity concentrations (Bq/kg) of radionuclides from ^238^U series, ^235^U and

^232^Th in pit lake sediments determined by alpha spectrometry. **Page S8**

**Table S7.** Distribution coefficient (K_d_ in kg_water_ / kg_sediment_) for radionuclides in pit lakes.

**Page S8**

**34 Pictures** from the sampling sites. **Pages S9-S17**

**Table S1**

Coordinates, size of the pit lake, working time and main ores mined in the selected sites. Nd= Not available data. Originally, database about pit lakes comes from: www.dykarna.nu (diving forum) and www.mindat.org – (database about localities, deposits, and mines worldwide).

| Site (label) | Coordinates (WGS84) | Size (m) 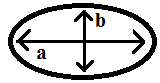 | Operation time | Extracted ore |
| --- | --- | --- | --- | --- |
| Site 1 (S1) | 58.91561, 16.30866 | 220x52 | 1936 - 1960 | Limestone |
| Site 2 (S2) | 58.972854, 17.715688 | A : 300x150 | 1850 - 1975 | Limestone |
|  |  | B: 30x20 |  |  |
| Site 3 (S3) | 59.570445, 15.821115 | A: 130x90 | 1870 - 1987 | Feldspar, quartz and Be. |
|  |  | B: 70x60 |  |  |
|  |  | C: 60x40 |  |  |
| Site 4 (S4) | 59.404341, 16.398146 | A: 500x200 | Nd | Granite |
|  |  | B: 100x50 |  |  |
| Site 5 (S5) | 59.327031, 15.464164 | A: 65x15 | Nd | Marble |
|  |  | B: 40x15 |  |  |
| Site 6 (S6) | 59.346274, 15.424268 | 120x20 | Nd | Marble |
| Site 7 (S7) | 59.336206, 17.675465 | 75x65 | 1884-1937 | Granite |
| Site 8 (S8) | 59.370036, 15.241238 | A: 290x220 | 1923-1975 | Limestone |
|  |  | B: 350 x 250 |  |  |
| Site 9 (S9) | 59.246169, 14.922706 | 210x92 | Nd | Limestone |
| Site 10 (S10) | 59.82793, 14.142001 | A: 50x50 | 1858-1965 | Fe |
|  |  | B: 95x30 |  |  |
| Site 11 (S11) | 59.323631, 15.460104 | A: 140x50 | Ongoing | Dolomite marble |
|  |  | B: 100x25 |  |  |
| Site 12 (S12) | 60.134115, 18.638993 | 32x24 | 1800-1905 | Fe, Zn, Cu, Pb |
| Site 13 (S13) | 60.148921, 15.303059 | 40x10 | 1873-1918 | Fe |
| Site 14 (S14) | 60.347778, 15.585278 | 60x15 | 1483-1920 | Au, Pb, Fe, Zn |
| Site 15 (S15) | 60.361111, 15.790556 | 114x83 | 1388-1967 | Magnetite |
| Site 16 (S16) | 60.080558, 15.939918 | A: 150x 50 | 1830-1968 | Fe |
|  |  | B: 30x30 |  |  |
| Site 17 (S17) | 56.360488, 14.344859 | 110x90 | Ongoing | Granite |
| Site 18 (S18) | 56.442611, 14.354317 | 600x40 | Nd | Granite |
| Site 19 (S19) | 55.659130, 13.402192 | 180x108 | 1893-1950 | Slate quarry |
| Site 20 (S20) | 56.395868, 14.251765 | 900x50 | Nd -1989 | Diabase |
| Site 21 (S21) | 56.168894, 14.362397 | 110x100 | Ongoing | Red Marble |
| Site 22 (S22) | 56.154134, 14.357090 | 180x70 | Nd | Granite |
| Site 23 (S23) | 56.151372, 14.372980 | 160x120 | Nd | Granite |

**Table S2**

Physico-Chemical parameters in Pit lakes water samples from Southern Sweden. Blank boxes: not available data on the multiparametric probe.

| Sample | pH | ORP(mV) | C(μS/cm) | DO (mg/L) | TDS (ppm) |
| --- | --- | --- | --- | --- | --- |
| WS01A | 8.26 | 12.7 | 258 | 12.3 |  |
| WS02A | 8.33 | 40 | 230 |  | 142 |
| WS02B | 7.8 | 71 | 316 |  | 193 |
| WS03A | 7.5 | 84 | 120 | 10.0 |  |
| WS03B | 7.07 |  | 97 | 8.5 |  |
| WS03C | 7.23 |  | 163 | 9.4 |  |
| WS04A | 7 | 269 | 576 | 10.6 |  |
| WS04B | 7.7 | 301 | 597 | 11.3 |  |
| WS05A | 8.42 | 68 | 300 | 11.5 |  |
| WS05B | 8.1 | 26 | 341 | 10.6 |  |
| WS06A | 7.95 | 222 | 350 | 11.4 |  |
| WS07A | 7.71 | 67 | 103 | 10.8 |  |
| WS08A | 8.29 | 42 | 298 |  | 199 |
| WS08B | 8.32 | 41 | 367 |  | 223 |
| WS09A | 7.98 | 28 | 391 | 10.6 |  |
| WS10A | 7.74 | 101 | 62 | 8.7 |  |
| WS10B | 6.96 | 99 | 47 | 7.9 |  |
| WS10C | 6.88 | 85 | 61 | 8.2 |  |
| WS11A | 8.27 | 31 | 431 |  | 304 |
| WS11B | 8.43 | 34 | 480 |  | 361 |
| WS12A | 8.12 | 53 | 532 |  | 327 |
| WS12B | 7.5 | 87 | 187 |  | 100 |
| WS13A | 7.06 | 47 | 205 | 7.8 |  |
| WS14A | 4.94 | 251 | 293 | 8.6 |  |
| WS15A | 7.5 | 157 | 211 | 11.5 |  |
| WS16A | 8.45 | 38 | 388 |  | 254 |
| WS17A | 7.9 | 69 | 317 |  | 153 |
| WS17B | 8.34 | 40 | 270 |  | 166 |
| WS18 | 7.97 | 61 | 193 |  | 110 |
| WS19A | 7.92 | 96 | 175 | 10.4 |  |
| WS20 | 7.46 | 89 | 130 |  | 79 |
| WS21 | 5.93 | 175 | 198 |  | 121 |
| WS22 | 6.85 | 114 | 107 | 8.7 |  |
| WS23 | 6.39 | 129 | 71 | 9.6 |  |

|  | mg/kg | | | | | | | μg/kg | | | | | | | | | ng/kg |
| --- | --- | --- | --- | --- | --- | --- | --- | --- | --- | --- | --- | --- | --- | --- | --- | --- | --- |
| Sample | Na | Mg | P | S | K | Ca | Fe | Mn | Cr | Cu | Zn | As | Sr | Ba | Pb | U | Th |
| WS01A | 10.7 | 15.0 | 2.25 | 322 | 32.5 | 177 | 0.084 | 29.1 | 0.21 | 13.5 | 659 | 2.68 | 262 | 59.8 | 29.7 | 0.37 | ND |
| WS02A | 9.8 | 9.1 | ND | ND | 7.1 | 112 | ND | 0.53 | ND | 2.00 | 346 | 1.10 | 182 | 16.8 | 2.47 | 14.4 | 70 |
| WS02B | 10.2 | 11.6 | ND | 307 | 11.3 | 210 | ND | 3.08 | ND | 8.1 | 196 | 1.47 | 131 | 22.0 | 4.94 | 10.1 | ND |
| WS03A | 15.7 | 7.4 | 4.43 | 591 | 22.7 | 74.0 | 0.830 | 10.5 | 0.56 | 8.8 | 480 | ND | 63.7 | 22.5 | 11.5 | 13.9 | ND |
| WS03B | 8.9 | 5.1 | ND | 128 | 13.2 | 32.9 | 0.713 | 36.0 | 2.99 | 4.9 | 96 | 0.38 | 54.2 | 9.8 | 21.3 | 11.7 | 110 |
| WS03C | 7.3 | 6.8 | 9.2 | 637 | 19.7 | 110 | 0.858 | 19.1 | 1.88 | 17.1 | 534 | 1.53 | 62.4 | 22.0 | 36.3 | 53.5 | ND |
| WS04A | 118 | 50.0 | ND | 316 | 45.5 | 276 | 0.350 | 22.7 | 4.52 | 29.3 | 399 | ND | 285 | 26.0 | 13.0 | 43.8 | ND |
| WS04B | 128 | 46.7 | ND | 328 | 43.6 | 382 | 0.277 | 20.0 | 1.74 | 69 | 435 | ND | 309 | 24.2 | 167.8 | 51.4 | 40 |
| WS05A | 16.2 | 46.4 | ND | ND | 16.9 | 272 | 0.275 | 15.2 | 0.90 | 27.8 | 446 | 1.15 | 103 | 11.2 | 15.5 | 0.64 | 70 |
| WS05B | 10.9 | 45.8 | 4.0 | 250 | 28.2 | 226 | 0.218 | 52.4 | 3.41 | 25.5 | 806 | ND | 81.7 | 11.2 | 17.0 | 0.07 | ND |
| WS06A | 7.2 | 74.9 | 4.0 | 628 | 27.6 | 162 | 0.706 | 13.1 | 4.24 | 12.9 | 480 | 0.77 | 82.2 | 15.0 | 10.9 | 2.32 | ND |
| WS07A | 8.2 | 5.1 | ND | ND | 15.1 | 66.7 | 0.644 | 4.38 | 2.57 | 9.6 | 140 | ND | 96 | 20.0 | 5.3 | 1.05 | ND |
| WS08A | 20.4 | 14.8 | 4.4 | 502 | 20.8 | 294 | 0.287 | 14.6 | 3.20 | 33.9 | 633 | 0.77 | 181 | 18.3 | 15.3 | 8.2 | ND |
| WS08B | 20.8 | 18.6 | ND | 199 | 9.2 | 181 | 4.3·10^-5^ | 1.00 | ND | 5.6 | 332 | 2.20 | 150 | 6.1 | 6.2 | 22.9 | ND |
| WS09A | 48.2 | 7.1 | ND | 400 | 17.5 | 225 | 0.366 | 6.7 | 1.18 | 11.4 | 561 | 2.30 | 222 | 33.1 | 12.5 | 1.65 | ND |
| WS10A | 5.59 | 4.1 | 2.96 | ND | 11.2 | 39.6 | 1.10 | 39.8 | 2.43 | 13.3 | 565 | ND | 36.6 | 12.7 | 15.5 | 0.34 | 39 |
| WS10B | 6.17 | 4.0 | 5.7 | 544 | 17.7 | 46 | 0.927 | 21.2 | 4.31 | 13.6 | 659 | ND | 33.1 | 19.8 | 15.1 | 0.22 | 190 |
| WS10C | 6.46 | 4.7 | 1.74 | 19.1 | 12.2 | 39.7 | 0.460 | 37.3 | 3.06 | 13.7 | 595 | ND | 32.9 | 15.2 | 19.5 | 0.15 | ND |
| WS11A | 31.4 | 100 | ND | 50.4 | 11.1 | 240 | 5.3·10^-3^ | 23.8 | 4.13 | 2.5 | 127 | 4.20 | 200 | 8.0 | 4.08 | 4.12 | ND |
| WS11B | 23.1 | 76.6 | 1.40 | 493 | 38.8 | 156 | ND | 4.03 | 0.93 | 13.7 | 418 | 2.94 | 145 | 18.6 | 7.2 | 7.7 | ND |
| WS12A | 41.1 | 20.5 | 1.73 | 357 | 20.4 | 243 | ND | 1.69 | 0.17 | 1.85 | 1536 | 0.73 | 168 | 15.1 | 5.78 | 2.29 | ND |
| WS12B | 6.9 | 3.75 | ND | 16.6 | 7.5 | 115 | ND | 5.08 | ND | 25.1 | 8436 | 1.10 | 40.9 | 20.1 | 34.5 | 38.3 | 37 |
| WS13A | 3.96 | 19.7 | 1.29 | ND | 13.3 | 113 | ND | 233 | ND | 11.7 | 316 | ND | 47.9 | 16.3 | 6.1 | 0.07 | ND |

**Table S3**

Elemental composition (mayor and traces) by ICP-MS in Pit lakes water samples from Southern Sweden. Uncertainty is around 20% with k=1. ND=Below Detection Limit

**Table S3 Continuation**

Elemental composition (mayor and traces) by ICP-MS in Pit lakes water samples from Southern Sweden. Uncertainty is around 20% with k=1

|  | mg/kg | | | | | | | μg/kg | | | | | | | | | ng/kg |
| --- | --- | --- | --- | --- | --- | --- | --- | --- | --- | --- | --- | --- | --- | --- | --- | --- | --- |
| Label | Na | Mg | P | S | K | Ca | Fe | Mn | Cr | Cu | Zn | As | Sr | Ba | Pb | U | Th |
| WS14A | 7.8 | 19.5 | ND | 73.9 | 9.2 | 93.4 | ND | 435 | ND | 71.8 | 5867 | ND | 89.0 | 20.6 | 691 | 0.34 | ND |
| WS15A | 10.0 | 15.5 | 0.63 | ND | 15.9 | 110 | 0.708 | 8.7 | 0.83 | 15.2 | 595 | ND | 220 | 14.4 | 7.6 | 55.4 | ND |
| WS16A | 34.0 | 29.7 | ND | 317 | 33.61 | 150 | 2.3·10^-4^ | 1.47 | 0.17 | 11.1 | 369 | 1.82 | 144 | 201 | 4.81 | 8.1 | ND |
| WS17A | 39.4 | 32.8 | 1.70 | ND | 38.2 | 104 | 5.5·10^-3^ | 84.6 | 17.8 | 50.3 | 286 | 0.73 | 175 | 19.4 | 6.5 | 0.92 | 360 |
| WS17B | 26.0 | 16.1 | 0.23 | ND | 24.1 | 69.7 | ND | 16.3 | 5.9 | 113 | 269 | 2.18 | 131 | 8.4 | 4.9 | 0.88 | ND |
| WS18 | 25.9 | 12.7 | 2.11 | 187 | 31.9 | 55.4 | 3.9·10^-3^ | 51.1 | 16.1 | 14.3 | 225 | 0.73 | 77.7 | 22.5 | 5.7 | 1.19 | 750 |
| WS19A | 25.7 | 17.4 | ND | ND | 23.9 | 58.0 | 0.873 | 15.8 | 1.60 | 9.3 | 131 | ND | 81.6 | 9.0 | 5.5 | 0.07 | ND |
| WS20 | 18.5 | 7.8 | ND | 193 | 9.9 | 48.0 | 0.001 | 17.2 | 3.08 | 46.2 | 373 | ND | 101 | 14.6 | 3.77 | ND | ND |
| WS21 | 31.0 | 6.4 | 4.53 | 626 | 20.4 | 63.7 | 0.001 | 509 | 1.78 | 29.6 | 418 | ND | 91.3 | 14.7 | 7.6 | 107 | 68 |
| WS22 | 0.10 | ND | 0.66 | 3.62 | 1.72 | 12.6 | 3.7·10^-5^ | 11.5 | ND | ND | ND | ND | 74.7 | 5.72 | 55.6 | ND | ND |
| WS23 | 0.03 | ND | 0.12 | 0.99 | 0.85 | 7.7 | ND | 98.5 | ND | ND | 85.8 | ND | 50.7 | 3.43 | ND | ND | ND |

**Table S4**

U, Th, Po isotopes by alpha spectrometry in Pit lakes water samples from Southern Sweden. Uncertainty is shown with k=1.

|  | ^238^U series (mBq/kg) | | | | ^232^Th series (mBq/kg) |
| --- | --- | --- | --- | --- | --- |
| Sample | ^238^U | ^234^U | ^230^Th | ^210^Po | ^232^Th |
| W S1 | 4.1±0.5 | 4.4±0.5 | 0.9±0.3 | 0.8±0.2 | 0.5±0.2 |
| W S2A | 154±4 | 164±4 | <0.3 | 2.9±0.4 | <0.3 |
| W S2B | 112±6 | 125±7 | <0.3 | 3.2±0.4 | <0.3 |
| W S3A | 174±4 | 258±5 | 1.4±0.6 | 10.8±0.7 | 0.2±0.1 |
| W S3B | 163±3 | 238±5 | 11.0±0.7 | 21.7±1.2 | 0.5±0.1 |
| W S3C | 609±15 | 767±18 | 2.1±0.6 | 23.6±1.2 | 0.7±0.3 |
| W S4A | 680±25 | 844±30 | 2.0±0.6 | 2.5±0.3 | 0.8±0.2 |
| W S4B | 618±16 | 757±19 | 4.6±1.4 | 4.7±0.5 | 0.7±0.2 |
| W S5A | 6.9±0.6 | 7.8±0.6 | 0.1±0.1 | 1.9±0.3 | <0.1 |
| W S5B | 5.8±0.8 | 4.4±0.7 | <0.1 | 5.2±1.6 | 0.5±0.1 |
| W S6A | 31±2 | 40±2 | 3.3±0.9 | 1.8±0.3 | 2.0±1.3 |
| W S18A | 12±1 | 17.4±1.6 | 0.8±0.3 | 4.3±0.6 | 0.9±0.5 |
| W S8A | 126±8 | 147±10 | 0.6±0.3 | 2.1±0.4 | <0.1 |
| W S8B | 236±9 | 363±13 | 0.3±0.1 | 3.5±0.4 | <0.1 |
| W S9A | 23±1 | 23.4±1.2 | 3.8±0.7 | 2.7±0.4 | 2.4±0.5 |
| W S10A | 3.6±0.5 | 5.2±0.6 | 2.1±0.4 | 5.2±0.8 | 0.8±0.3 |
| W S10B | 2.7±0.5 | 3.9±0.6 | 1.5±0.3 | 3.7±0.5 | 1.0±0.3 |
| W S10C | 3.3±0.6 | 6.5±0.8 | 0.9±0.4 | 6.1±0.7 | 0.6±0.2 |
| 2W S11A | 63±4 | 77±5 | 0.1±0.1 | 1.4±0.5 | 0.1±0.1 |
| 2W S11B | 95±5 | 106±6 | 1.3±0.6 | 1.7±0.6 | 1.5±0.3 |
| W S12A | 25±2 | 30±2 | <0.4 | 3.7±0.4 | <0.3 |
| W S12B | 0.3±0.1 | 0.3±0.1 | <0.6 | 4.3±0.5 | <0.4 |
| W S13A | 1.4±0.4 | 2.3±0.4 | 1.4±0.7 | 5.9±0.9 | 0.5±0.2 |
| W S14A | 3.4±0.5 | 4.9±0.6 | 0.4±0.2 | 4.6±0.6 | 0.6±0.2 |
| W S15A | 735±26 | 1069±35 | 1.2±0.4 | 16.5±1.0 | 0.4±0.1 |
| W S16A | 80±4 | 144±6 | 0.2±0.1 | 4.0±0.6 | <0.4 |
| W S17A | 13±1 | 17.7±1.2 | <0.4 | 10.4±0.9 | <0.4 |
| W S17B | 4.0±0.6 | 4.8±0.6 | <0.4 | 3.6±0.3 | <0.3 |
| W S18A | 15.4±1.2 | 15.9±1.3 | <0.3 | 55±3 | <0.3 |
| W S19A | 1.4±0.3 | 1.9±0.4 | 0.7±0.4 | 1.6±0.3 | 0.2±0.1 |
| W S20A | 6.0±0.9 | 6.9±1.2 | 1.4±0.5 | 10.7±0.8 | 0.6±0.2 |
| W S21A | 1184±30 | 1701±43 | 26.3±3.3 | 95±4 | 8.8±2.7 |
| W S22A | 84±3 | 109±4 | 0.5±0.3 | 18.6±1.5 | 0.2±0.1 |
| W S23A | 66±2 | 91±3 | 0.6±0.2 | 22.8±1.8 | 0.1±0.1 |

**Table S5**

XRF elemental composition in Pit lakes sediment samples from Southern Sweden. N.D. = below detection limit~1-5 mg/kg.

| Sample | % | | | | | mg/kg | | | | | | | | |
| --- | --- | --- | --- | --- | --- | --- | --- | --- | --- | --- | --- | --- | --- | --- |
|  | SiO2 | Al2O3 | Fe2O3 | MnO | SO3 | As | Ba | Cr | Cu | Pb | Sr | Th | U | Zn |
| Sed S3A | 77 | 11.3 | 1.7 | 0.09 | 0.04 | 9.4 | 131 | 6.4 | N.D. | 30.4 | 48 | 18.9 | 9.1 | 70 |
| Sed S3A-2 | 71 | 12.7 | 3.6 | 0.13 | 0.12 | 5.1 | 207 | N.D. | 8.0 | 73 | 50 | 16.5 | 17.8 | 99 |
| Sed S3B | 61 | 16.0 | 3.6 | 0.08 | 0.25 | 12.9 | 207 | 20.3 | 7.1 | 58 | 38 | 20.8 | 30 | 124 |
| Sed S3B-2 | 59 | 16.0 | 4.7 | 0.10 | 0.27 | 6.0 | 215 | 17.2 | 17.3 | 71 | 48 | 19.3 | 35 | 146 |
| Sed S3C-2 | 71 | 12.3 | 3.1 | 0.08 | 0.28 | 13.4 | 266 | 16.8 | 16.6 | 45 | 68 | 19.3 | 24.2 | 105 |
| Sed S4A | 72 | 10.8 | 4.8 | 0.09 | 0.01 | 16.6 | 308 | 36 | N.D. | 19.7 | 117 | 21.8 | 8.4 | 83 |
| Sed S6A | 9.8 | 1.7 | 0.6 | 0.08 | 0.01 | 0.4 | 35 | N.D. | N.D. | 1.3 | 33 | 17.5 | 4.0 | 83 |
| Sed S9A | 68 | 9.5 | 3.4 | 0.08 | 0.08 | 1.8 | 401 | 73 | 16.4 | 15.4 | 139 | 17.8 | 6.0 | 93 |
| Sed S10A | 68 | 6.9 | 9.2 | 0.17 | 0.05 | 17.9 | 493 | 107 | 69 | 24.4 | 104 | 12.6 | 4.4 | 209 |
| Sed S11B | 36 | 4.2 | 1.2 | 0.07 | 0.02 | N.D. | 194 | N.D. | N.D. | 8.8 | 75 | 14.4 | 4.5 | 113 |
| Sed S14A | 64 | 7.7 | 9.1 | 0.06 | 0.65 | 52 | 371 | 40 | 90 | 2032 | 83 | 17.7 | 5.0 | 748 |
| Sed S14A-2 | 69 | 8.5 | 8.2 | 0.05 | 0.97 | 20.0 | 422 | 34 | 105 | 1851 | 164 | 16.7 | 7.3 | 351 |
| Sed S15A | 65 | 12.8 | 9.7 | 0.05 | 0.02 | 4.7 | 863 | 97 | N.D. | 6.0 | 33 | 17.1 | 4.7 | 65 |
| Sed S19A | 80 | 4.0 | 1.3 | 0.05 | 0.02 | 14.2 | 170 | N.D. | N.D. | 11.6 | 85 | 18.9 | 4.9 | 71 |

**Table S6**

Activity concentrations (Bq/kg) of radionuclides from ^238^U series, ^235^U and ^232^Th in pit lake sediments determined by alpha spectrometry

|  | ^238^U series | | | | ^235^U | ^232^Th |
| --- | --- | --- | --- | --- | --- | --- |
| Sample | ^238^U | ^234^U | ^230^Th | ^210^Po |  |  |
| Sed S3A | 399±26 | 381±26 | 437±23 | 363±26 | 17±3 | 73±5 |
| Sed S3B | 1192±35 | 1390±40 | 1400±86 | 1685±110 | 48±3 | 112±9 |
| Sed S3C-2 | 895±29 | 914±33 | 950±80 | 890±45 | 31±2 | 105±10 |
| Sed S4A | 78±2 | 80±2 | 89±3 | 94±4 | 3.9±0.2 | 98±2 |
| Sed S6A | 4.7±0.4 | 4.2±0.4 | 13±2 | 15±1 | <0.1 | 11±2 |
| Sed S9A | 76±2 | 46±2 | 74±4 | 209±8 | 3.3±0.3 | 81±5 |
| Sed S10A | 35±1 | 33±2 | 49±3 | 48±2 | 1.3±0.2 | 50±2 |
| Sed S11B | 19±2 | 17±2 | 15±2 | 16±2 | 1.0±0.3 | 11±1 |
| Sed S14A | 23±1 | 23±1 | 31±1 | 57±7 | 0.9±0.4 | 25±1 |
| Sed S15A | 67±2 | 701±1 | 77±3 | 80±3 | 3.1±0.2 | 66±3 |
| Sed S19A | 22±1 | 24±1 | 52±1 | 35±2 | 1.2±0.1 | 35±2 |
| Sed S21A | 199±19 | 220±21 | 245±17 | 230±15 | 8.1±0.8 | 110±15 |

**Table S7**

Distribution coefficient (K_d_ in kg_water_ / kg_sediment_) for radionuclides in pit lakes.

| Site | ^238^U | ^234^U | ^230^Th | ^210^Po | ^232^Th |
| --- | --- | --- | --- | --- | --- |
| S3A | (1.93±0.07)·10^3^ | (1.30±0.05)·10^3^ | (2.35±0.99)·10^5^ | (2.77±0.19)·10^4^ | (2.12±0.43)·10^5^ |
| S3B | (9.01±0.39)·10^3^ | (6.18±0.27)·10^3^ | (1.33±0.16)·10^5^ | (6.24±0.41)·10^4^ | (3.23±0.72)·10^5^ |
| S3C | (1.36±0.05)·10^3^ | (1.08±0.04)·10^3^ | (4.03±1.29)·10^5^ | (4.08±0.22)·10^4^ | (2.01±0.41)·10^5^ |
| S4A | (1.13±0.04)·10^2^ | (9.11±0.32)·10^1^ | (3.89±1.24)·10^4^ | (3.27±0.36)·10^4^ | (9.37±1.87)·10^4^ |
| S6A | (2.40±0.66)·10^2^ | (1.86±0.51)·10^2^ | (2.28±0.69)·10^3^ | (7.52±1.69)·10^3^ | (3.66±0.85)·10^3^ |
| S9A | (2.55±0.40)·10^3^ | (2.49±0.39)·10^3^ | (1.53±0.34)·10^4^ | (3.41±0.60)·10^4^ | (1.45±0.29)·10^4^ |
| S10A | (9.24±1.53)·10^3^ | (6.41±0.93)·10^3^ | (1.59±0.34)·10^4^ | (8.94±1.52)·10^3^ | (3.92±0.82)·10^4^ |
| S11B | (1.85±0.37)·10^2^ | (1.65±0.33)·10^2^ | (1.31±0.56)·10^4^ | (1.21±0.47)·10^4^ | (9.83±2.38)·10^3^ |
| S14A | (6.18±1.03)·10^3^ | (4.26±0.64)·10^3^ | (5.51±2.34)·10^4^ | (1.12±0.15)·10^4^ | (5.41±1.17)·10^4^ |
| S15A | (9.87±1.22)·10^1^ | (6.79±0.83)·10^1^ | (5.99±2.19)·10^4^ | (5.15±0.35)·10^3^ | (1.82±0.37)·10^5^ |
| S19A | (1.44±0.36)·10^4^ | (1.05±0.22)·10^4^ | (2.77±1.43)·10^4^ | (1.83±0.34)·10^4^ | (1.20±0.40)·10^5^ |
| S21A | (2.01±0.16)·10^2^ | (1.40±0.11)·10^2^ | (9.07±1.45)·10^3^ | (2.30±0.23)·10^3^ | (1.85±0.41)·10^4^ |
| S22A | (1.28±0.07)·10^4^ | (9.82±0.50)·10^3^ | (2.18±1.33)·10^6^ | (3.84±0.31)·10^4^ | (2.38±0.50)·10^6^ |
| S23A | (7.79±0.30)·10^3^ | (5.62±0.20)·10^3^ | (8.52±3.12)·10^5^ | (2.28±0.18)·10^4^ | (1.80±0.37)·10^6^ |

Pictures of the Lakes in this work: (All Photos taken by Juan Mantero)


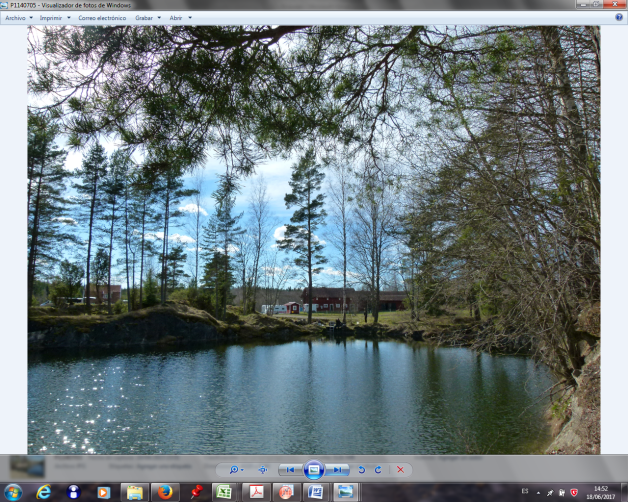

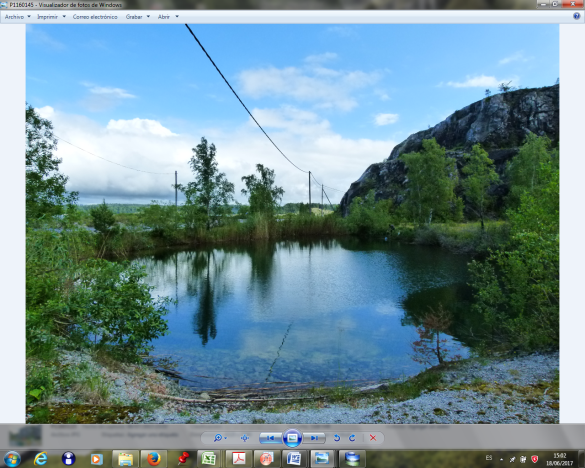


Picture 1: Site 1 Picture 2: Site 2B


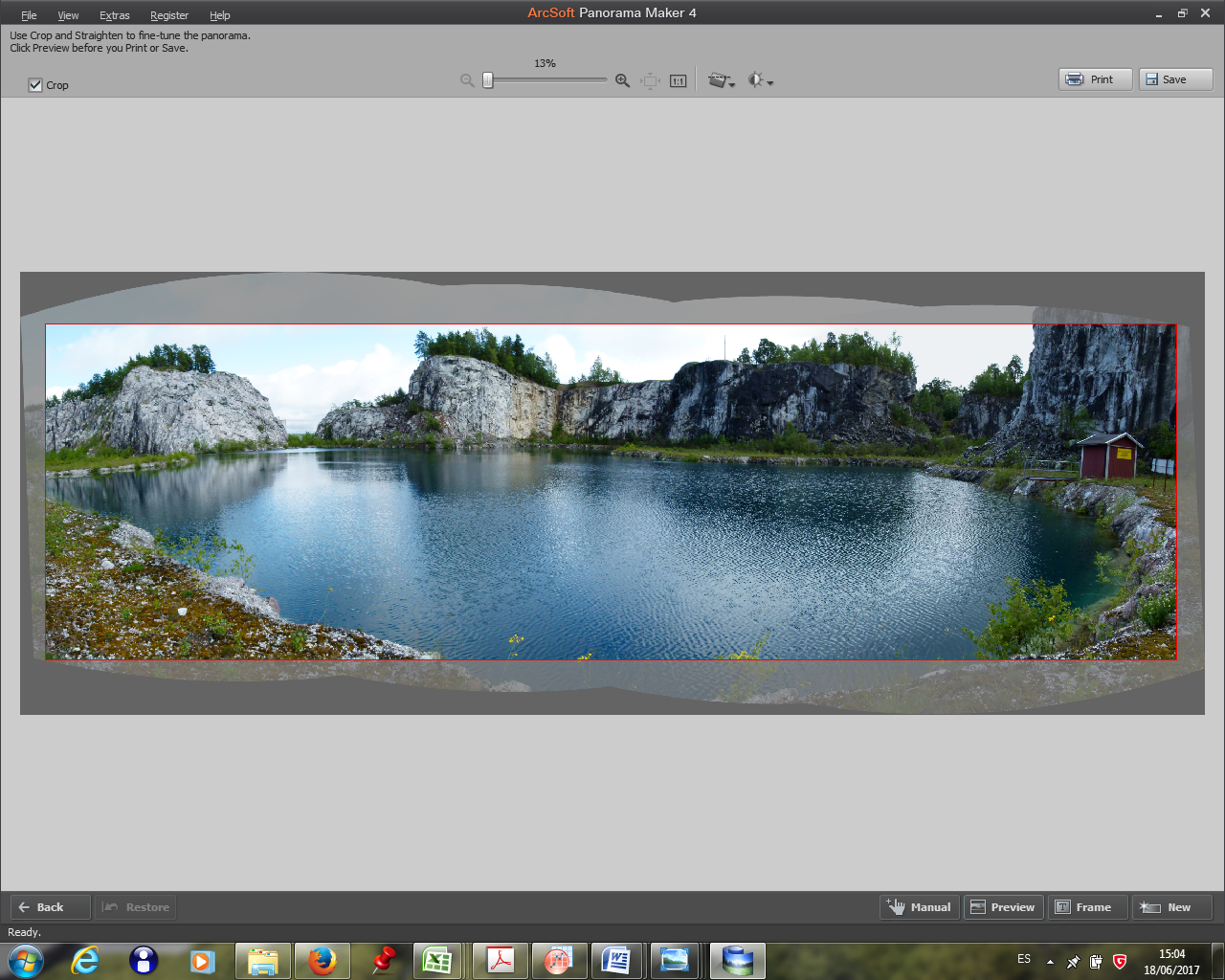


Picture 3: Site 2A


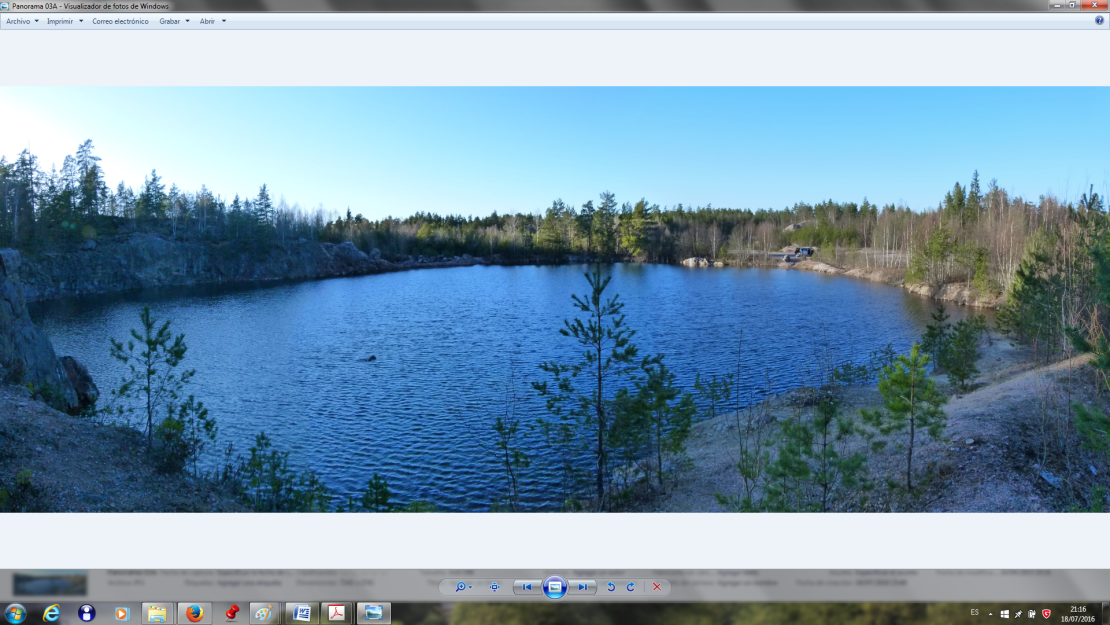


Picture 4: Site 3A


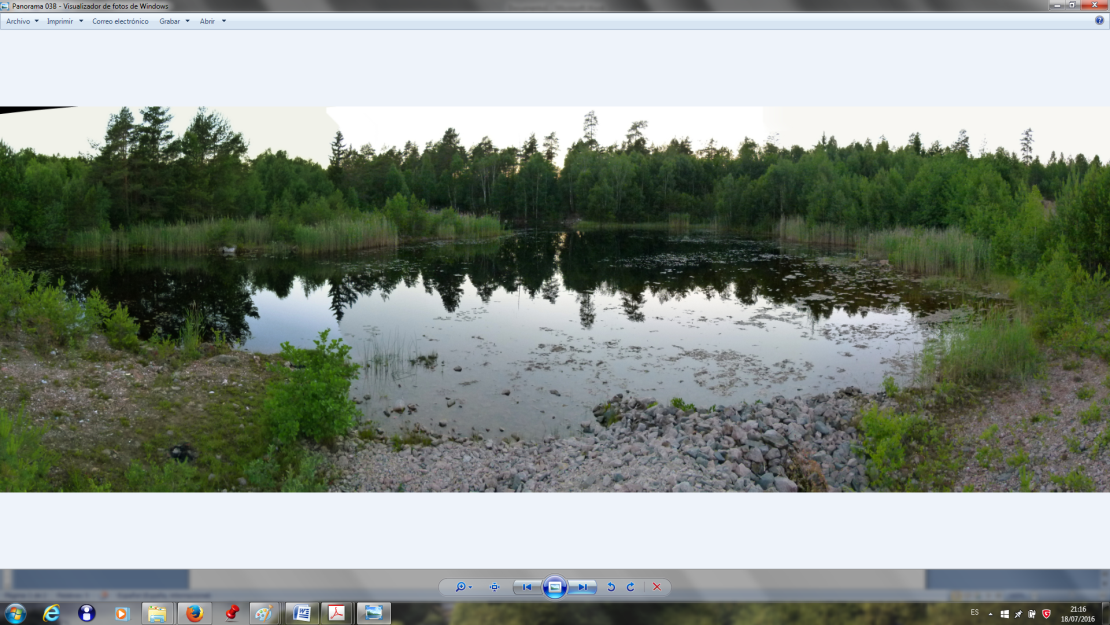


Picture 5: Site 3B


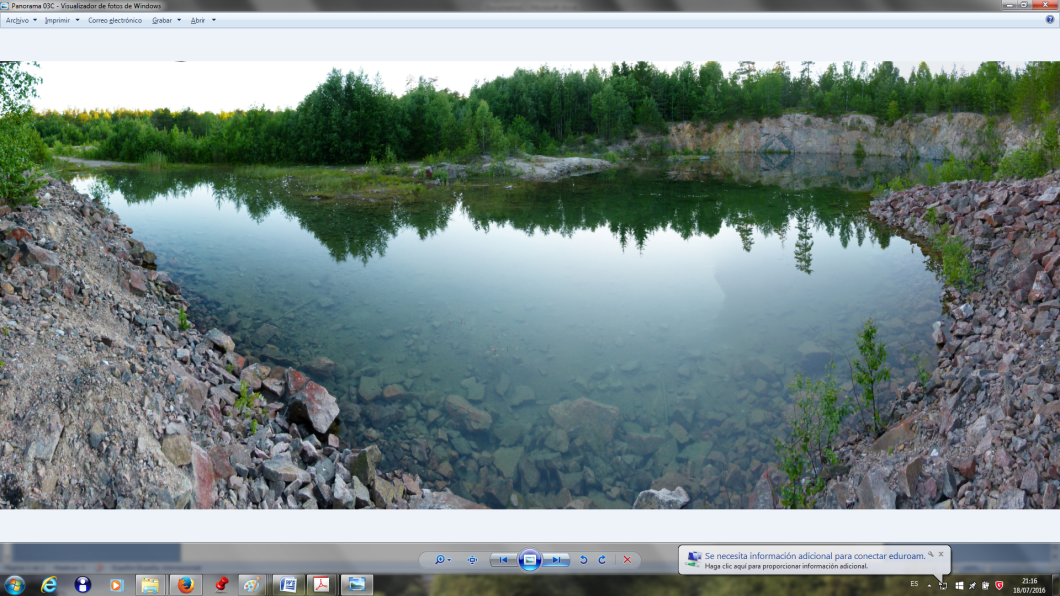


Picture 6: Site 3C


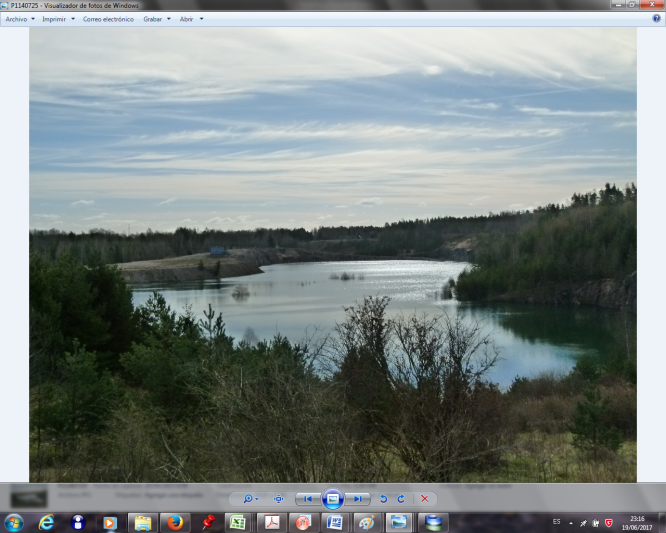

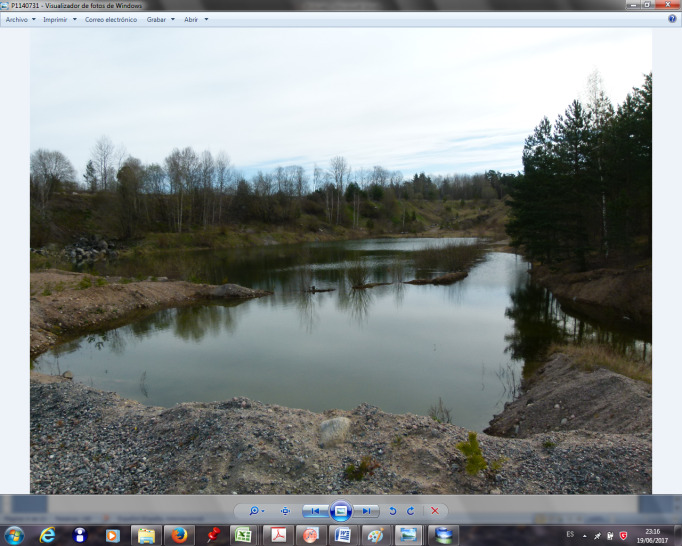


Picture 7: Site 4A Picture 8: Site 4B


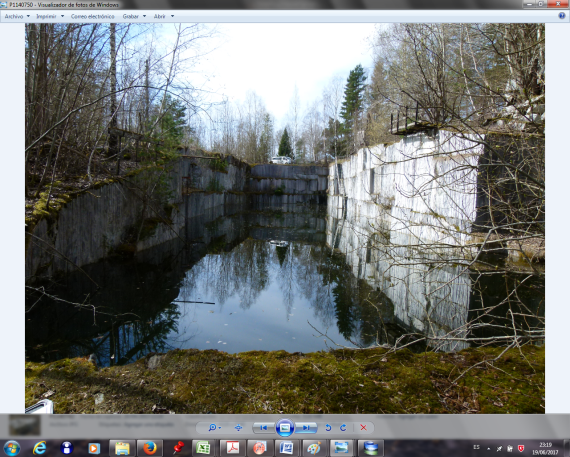

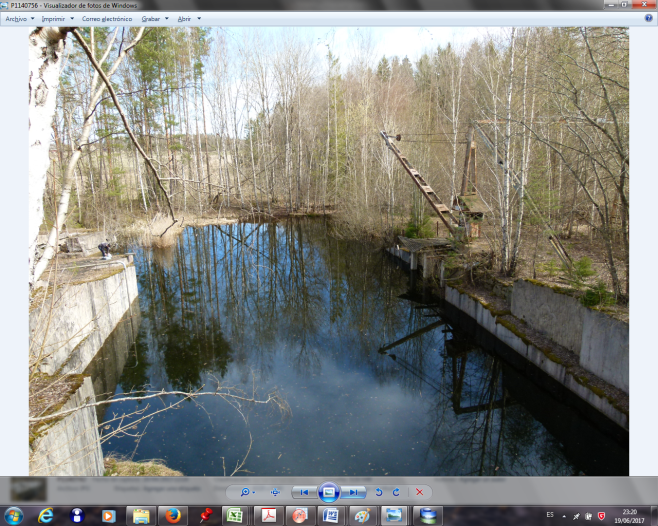


Picture 9: Site 5A Picture 10: Site 5B


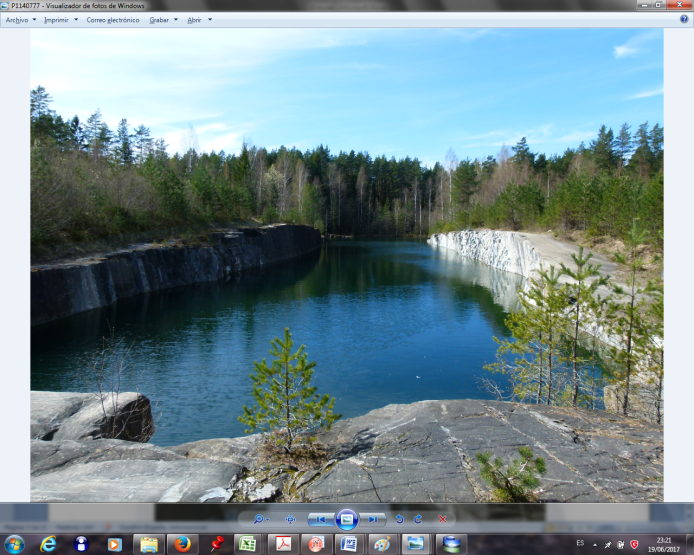


Picture 11: Site 6


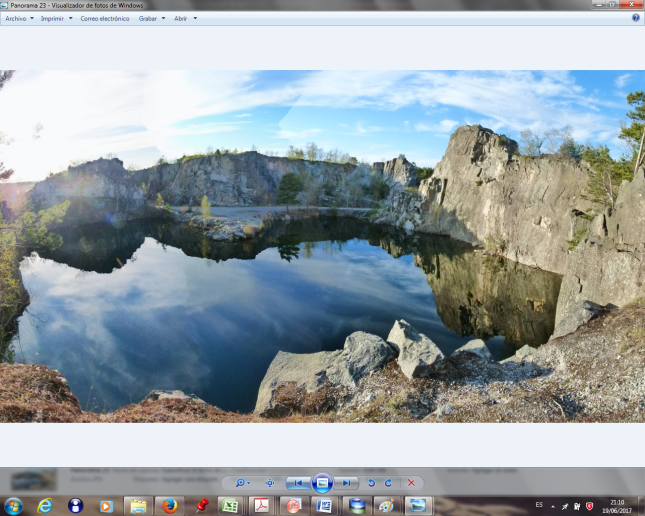


Picture 12: Site 7


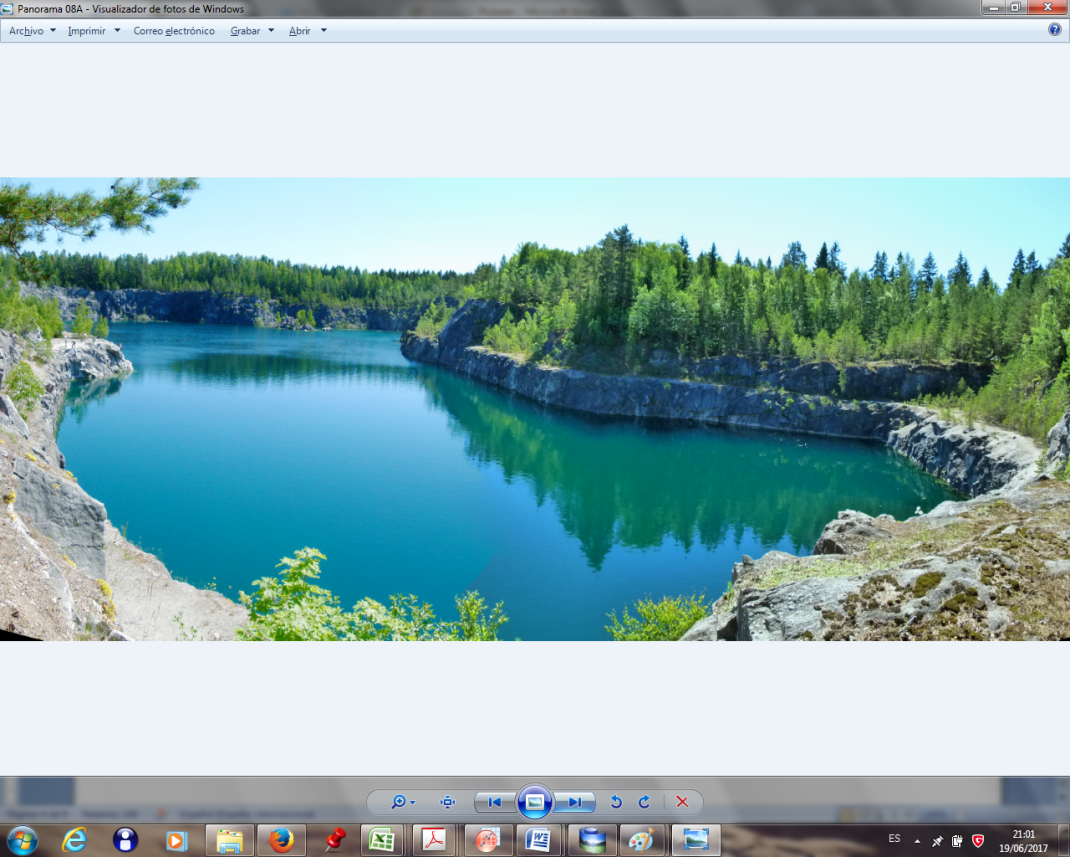


Picture 13: Site 8A


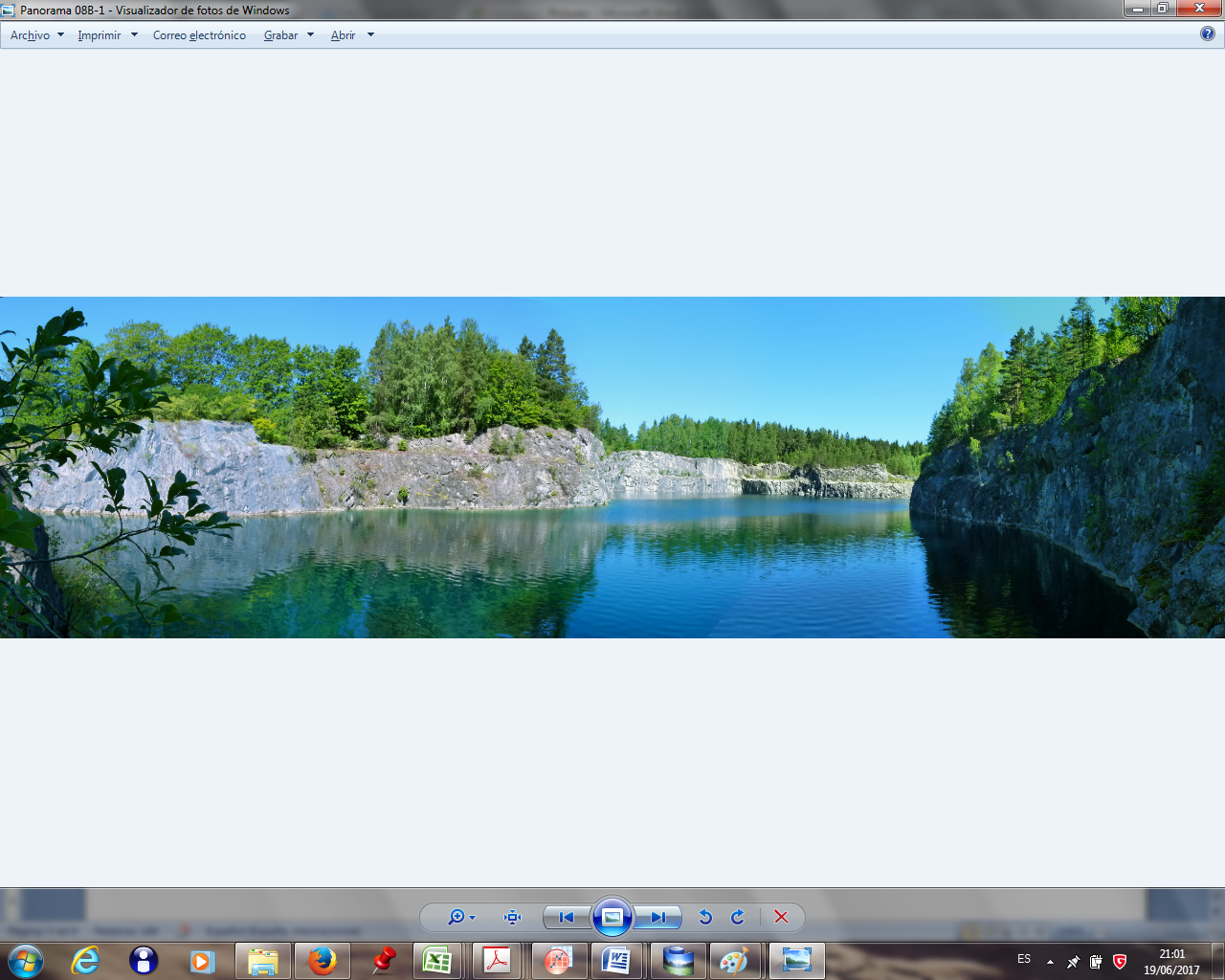


Picture 14: Site 8B


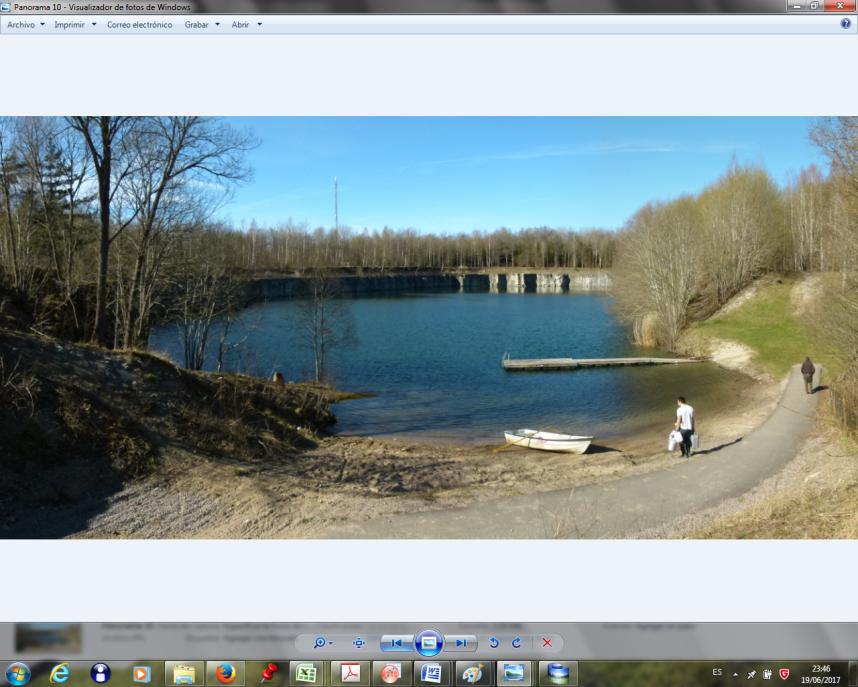


Picture 15: Site 9


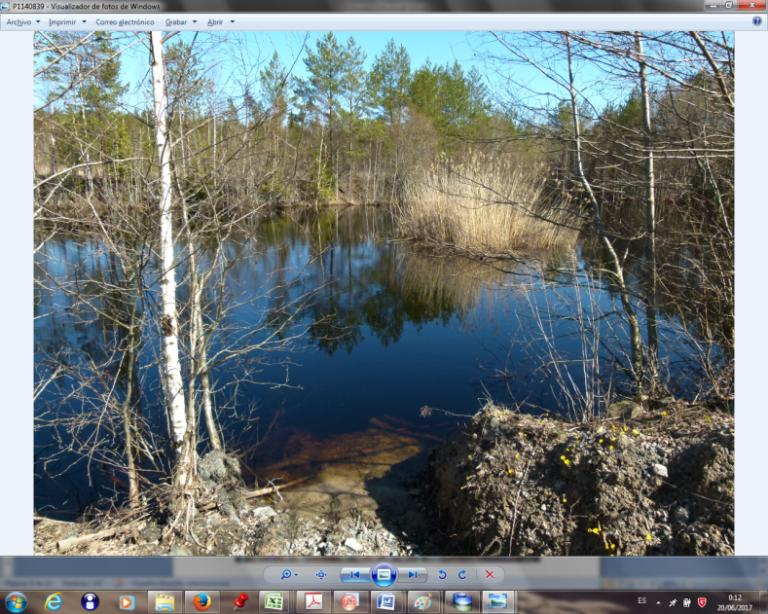

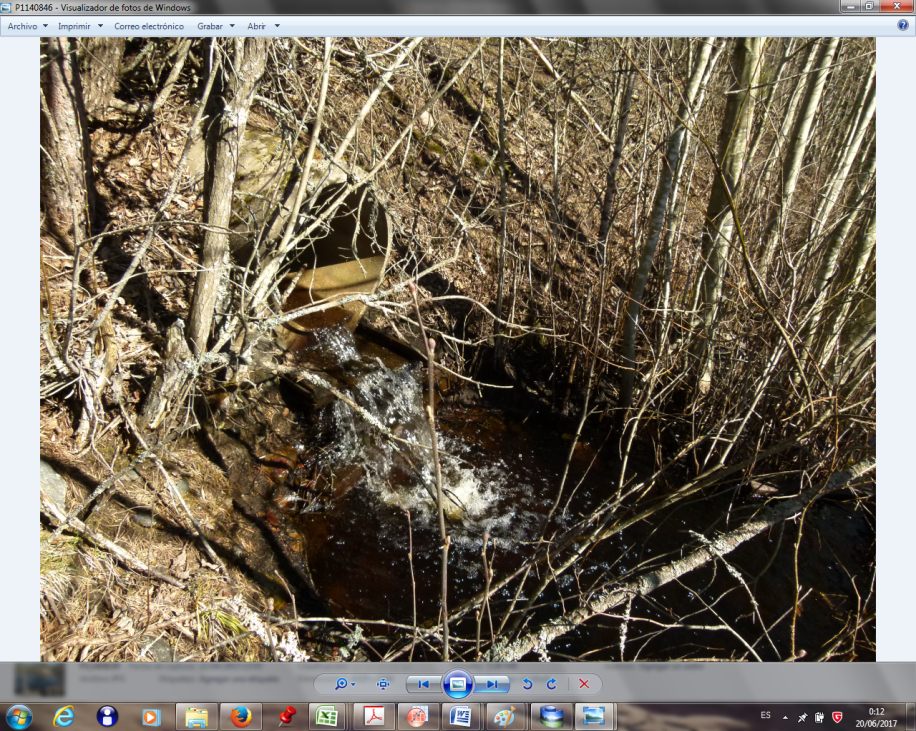


Picture 16: Site 10A Picture 17: Site 10B


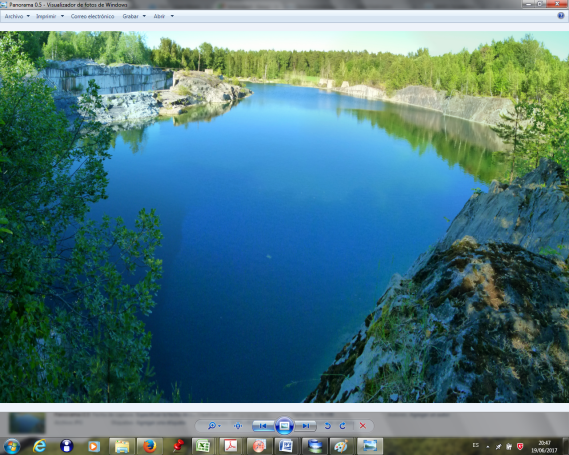

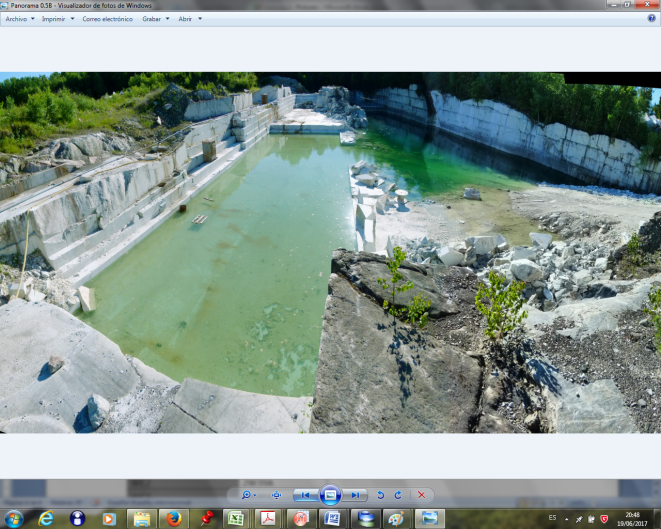


Picture 18: Site 11A Picture 19: Site 11B


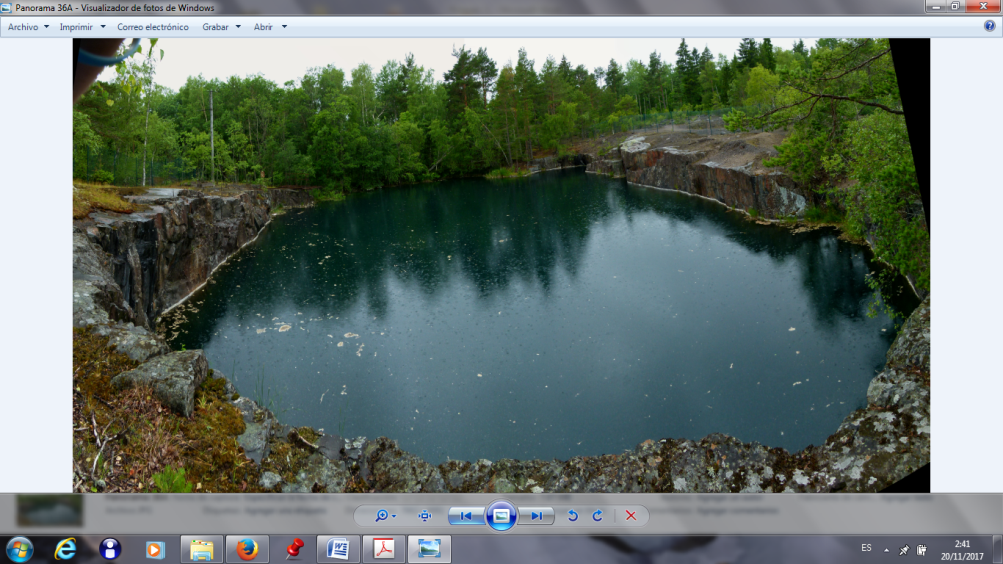

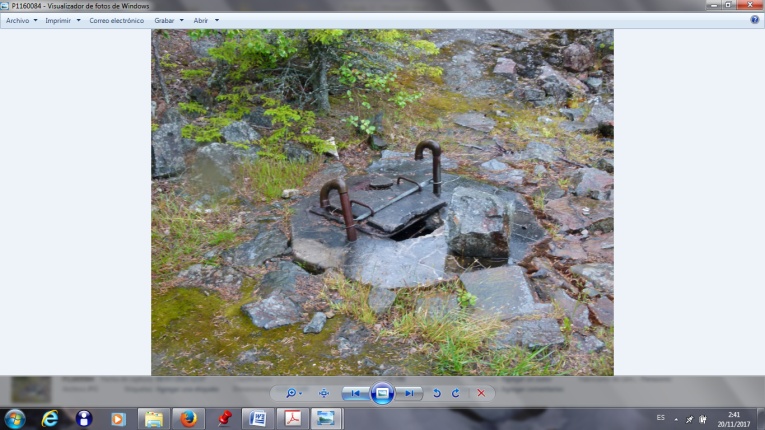


Picture 20: Site 12A Picture 21: Site 12B


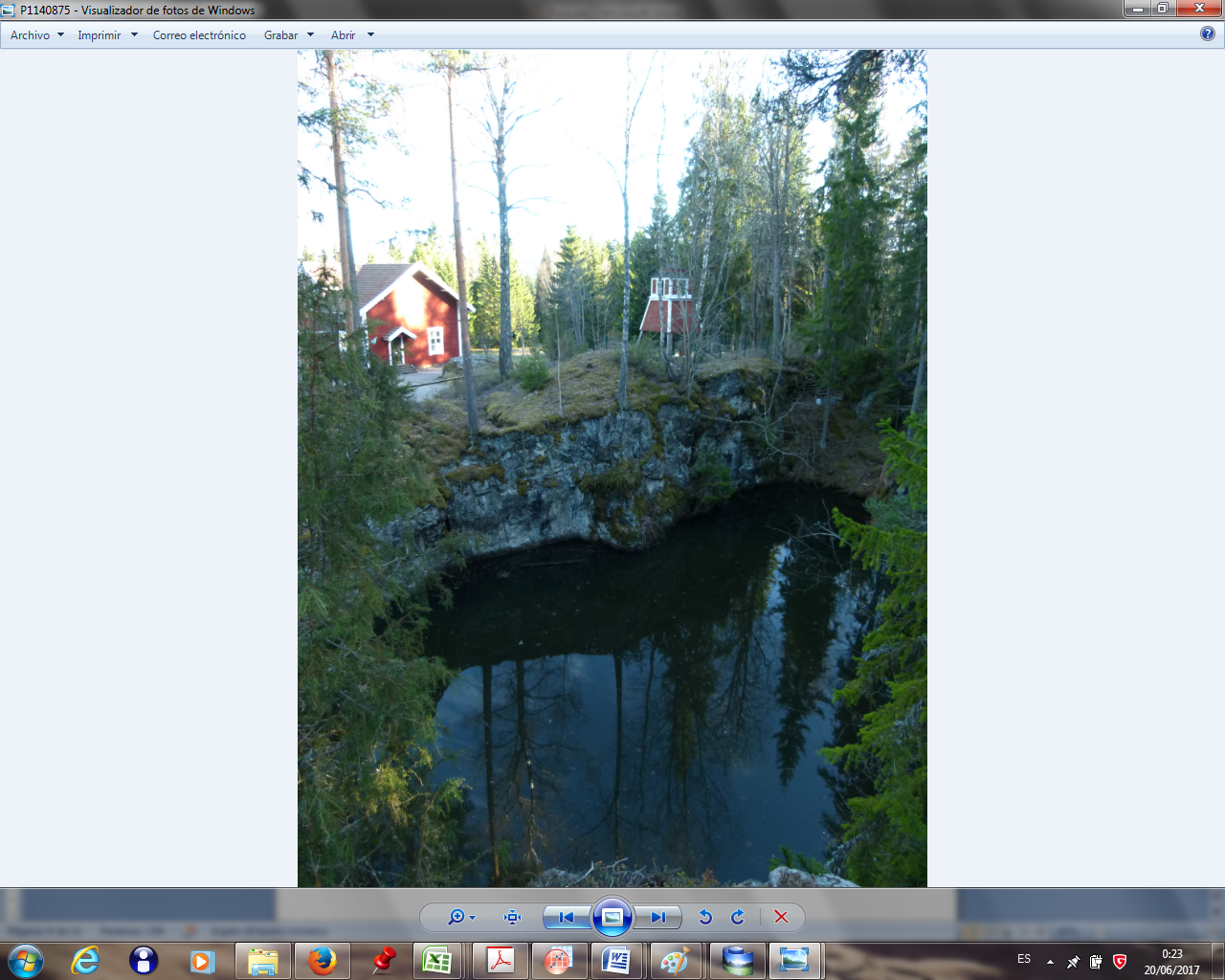


Picture 22: Site 13


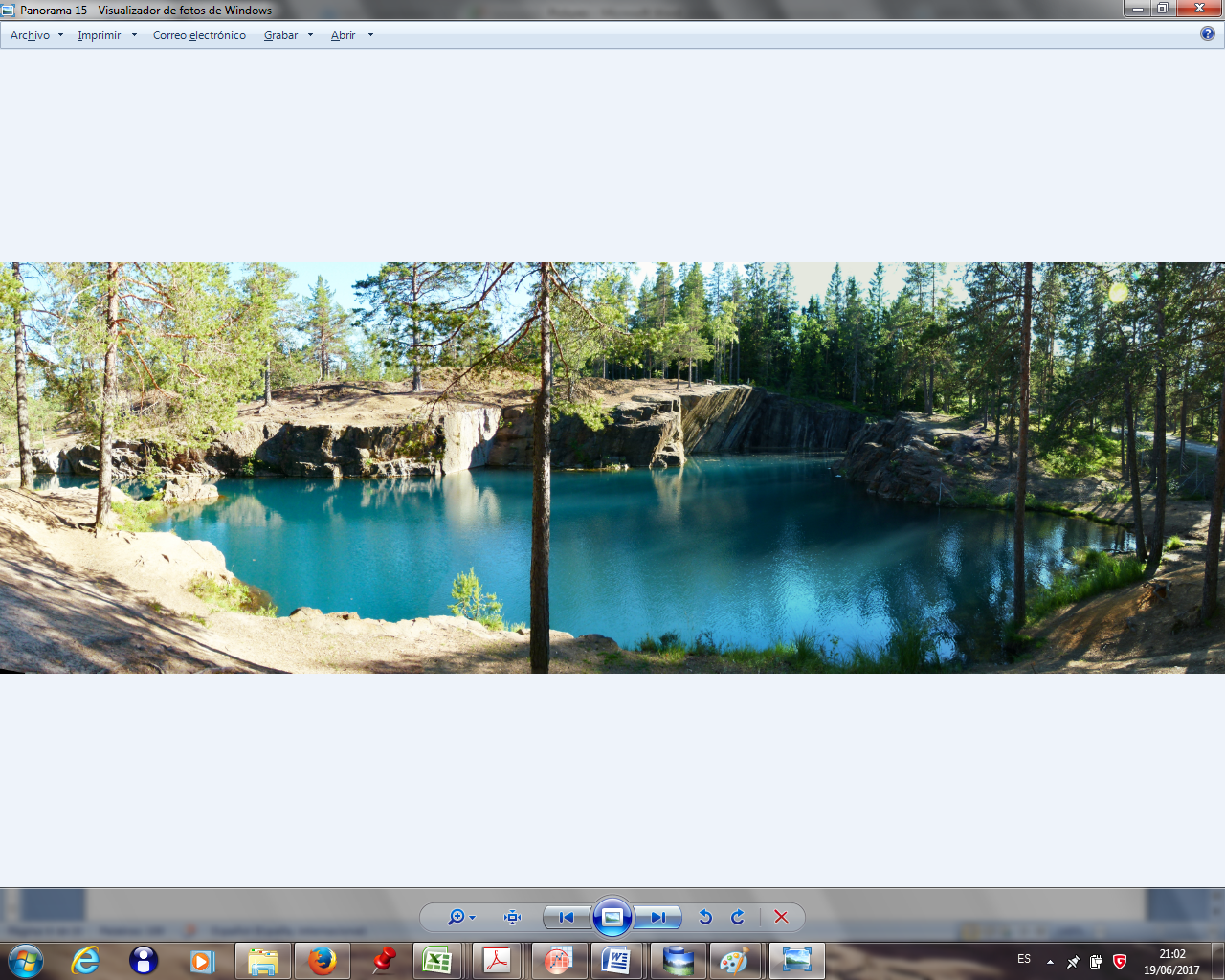


Picture 23: Site 14


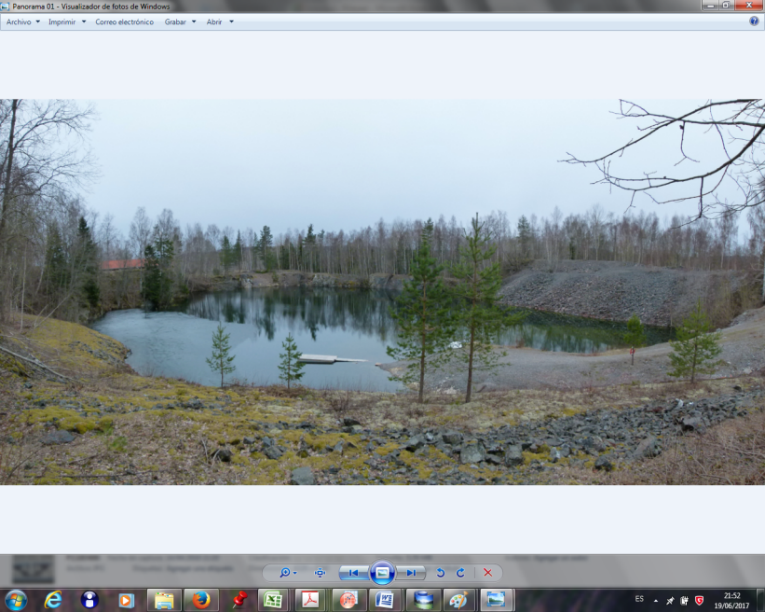


Picture 24: Site 15


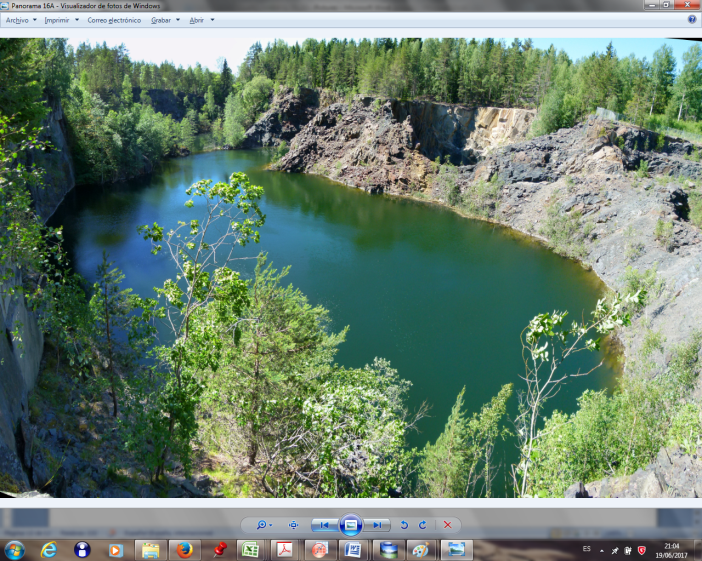


Picture 25: Site 16A


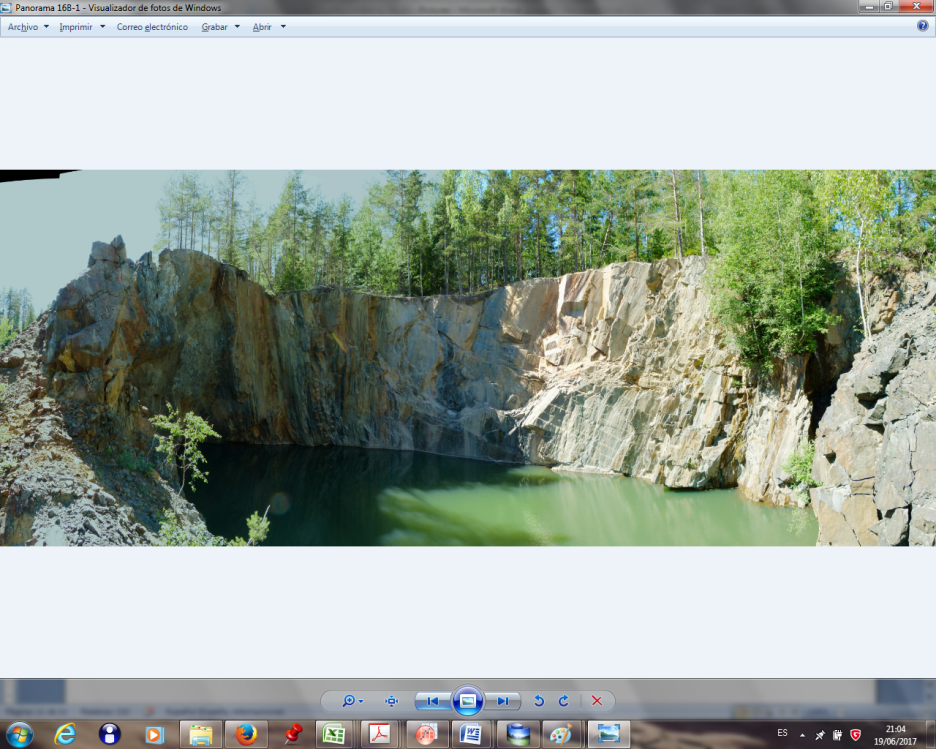


Picture 26: Site 16B


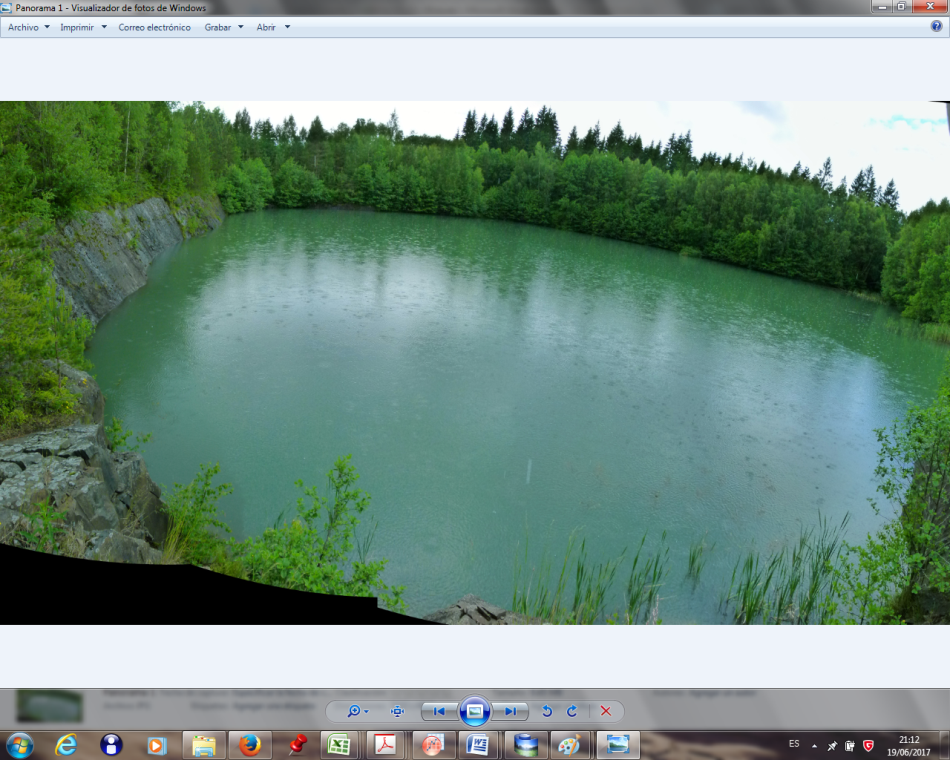

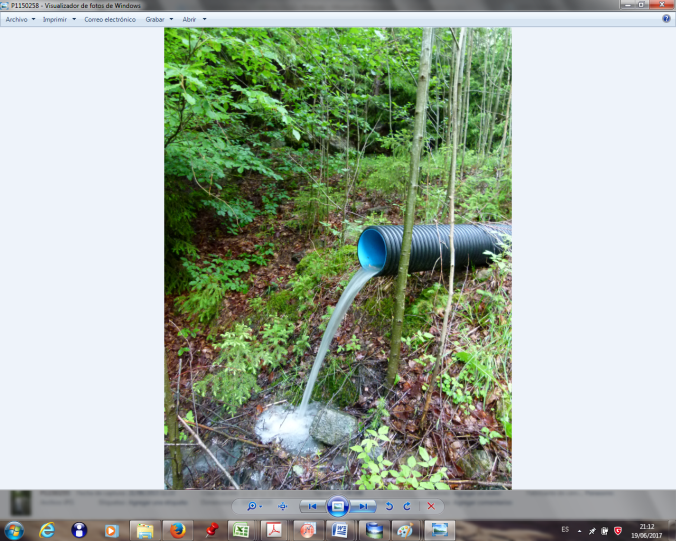


Picture 27: Site 17A Picture 28: Site 17B


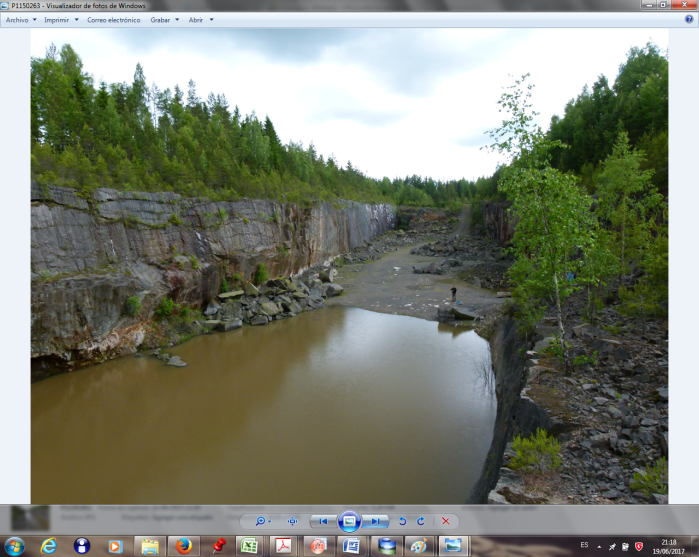

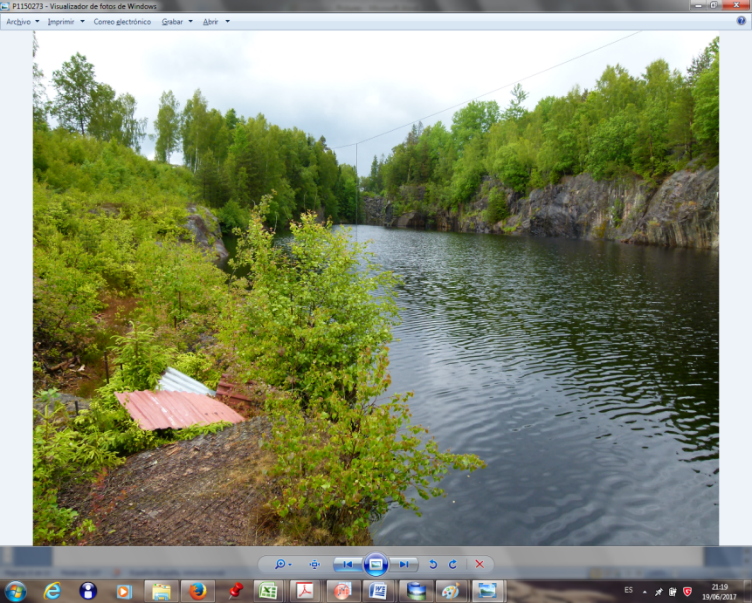


Picture 29: Site 18 Picture 30: Site 20


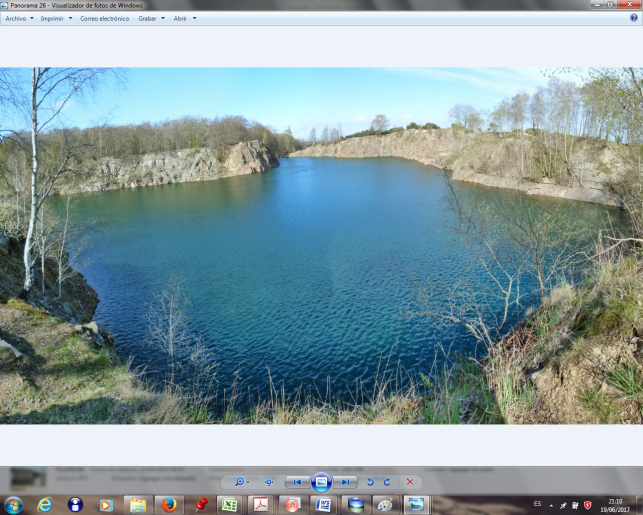


Picture 31: Site 19


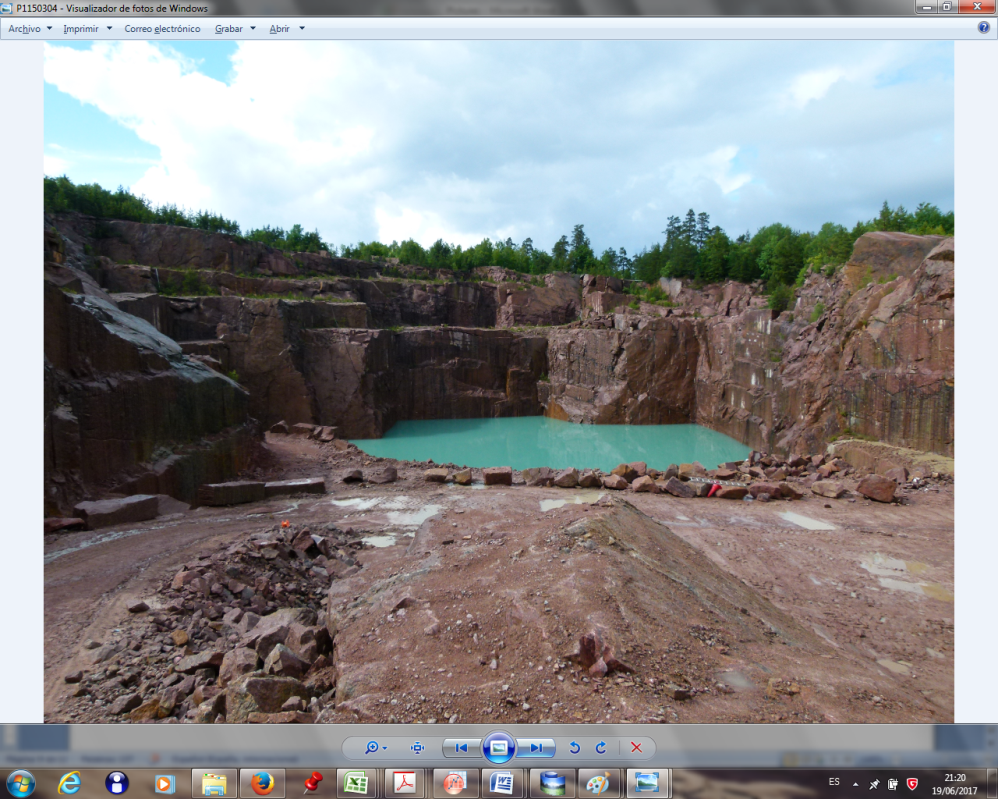


Picture 32: Site 21


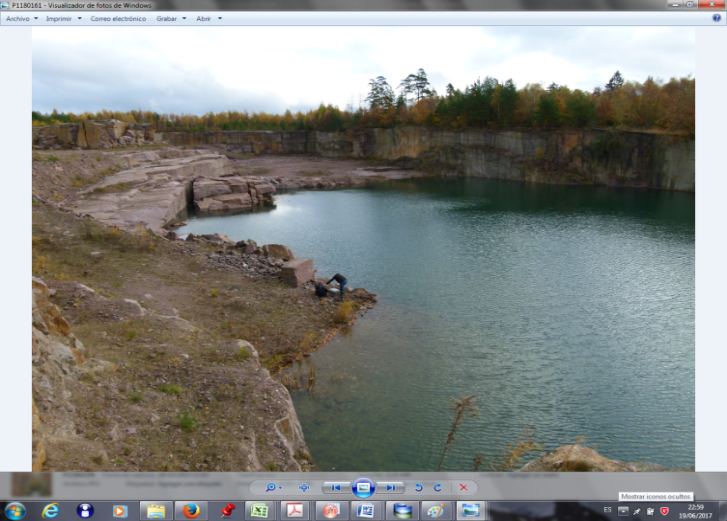

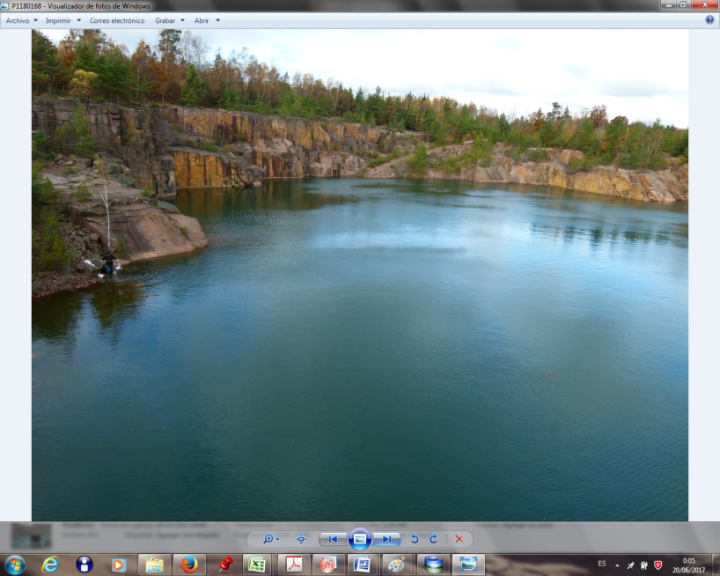


Picture 33: Site 22 Picture 34: Site
